# Different But Complementary Motor Functions Reveal an Asymmetric Recalibration of Upper Limb Bimanual Coordination

**DOI:** 10.1523/ENEURO.0112-25.2025

**Published:** 2026-01-02

**Authors:** Ada Kanapskyte, Jesus Alejandro Garcia Arango, Sanjay Joshi, Stephen K. Robinson, Jonathon S. Schofield, Lee M. Miller, Wilsaan M. Joiner, Weiwei Zhou

**Affiliations:** ^1^Departments of Biomedical Engineering, University of California Davis, Davis, California 95616; ^2^Neurobiology, Physiology and Behavior, University of California Davis, Davis, California 95616; ^3^Mechanical and Aerospace Engineering, University of California Davis, Davis, California 95616; ^4^Otolaryngology/Head and Neck Surgery, University of California Davis, Davis, California 95616; ^5^Neurology, University of California Davis, Davis, California 95616

## Abstract

Bimanual coordination, fundamental to human motor control, typically involves the execution of different functions by the two limbs (e.g., opening a jar). Previous research has largely investigated bimanual control through simple coordination tasks in which the limbs perform similar movements (e.g., finger tapping); however, few studies have specifically examined coordination when the two limbs perform different yet complementary functions. In the current study, participants performed point-to-point movements of a rectangular cursor, where one limb controlled cursor trajectory and the other rotated a knob to match a target orientation. Participants (*N* = 116, 76 female, 1 nonbinary; 92% right-handed) were divided into four groups and completed the task with a visual feedback gain perturbation (an increase or decrease) applied either to the cursor trajectory or orientation. Our results showed rapid adaptation to perturbations of visual feedback of the movement trajectory, affecting both the perturbed limb controlling the trajectory and the unperturbed limb controlling the orientation. Conversely, perturbation to the visual orientation feedback primarily only influenced the perturbed limb controlling orientation, with minimal impact on movement trajectory metrics. Importantly, these results were independent of reaching amplitude, duration, and limb dominance. In addition, we assessed the temporal coordination between the two limbs and found that perturbations in visual trajectory feedback led to significant changes in limb coordination, whereas no notable difference was observed for perturbations of orientation. These findings indicate asymmetries in bimanual motor recalibration dependent on the perturbed aspect of visual feedback (orientation vs trajectory), suggesting differences in underlying neural mechanisms and interhemispheric communication.

## Significance Statement

Bimanual coordination in daily life typically requires the upper limbs to perform different but complementary motor functions (e.g., opening a jar). There remains a gap in understanding how the two hands coordinate control to accomplish such tasks. To address this, we developed a novel bimanual coordination task that requires the two hands to jointly control different aspects of a single virtual object. We measured how perturbations of one feature of the task influenced the spatiotemporal properties of motions in the other unperturbed limb. Independent of limb dominance, we found asymmetries in the motor learning of our task that depended on the perturbed aspect of visual feedback (object orientation vs trajectory), suggesting differences in the underlying neural mechanisms and interhemispheric communication.

## Introduction

Motor learning is the process of attaining and refining performance on a motor task, from simple finger tapping movements to complex body motions (Krakauer et al., 2019). It encompasses both long-term improvements in motor performance and short-term adjustments to counteract unexpected changes in the environment, task, or body. Motor adaptation is a form of motor learning that involves rapid, short-term corrections in response to movement errors. These errors typically arise when the expected sensory feedback/consequences from movement differs from the actual feedback. In upper limb movements, examples of motor adaptation include adjusting the temporal patterns of the applied force to counter a physical perturbation during reaching (e.g., velocity- or position-dependent force field; Shadmehr and Mussa-Ivaldi, 1994; Diedrichsen and Gush, 2009; Sing et al., 2009; Yousif and Diedrichsen, 2012; Wu et al., 2014; Hosseini et al., 2017; Fitzgerald et al., 2024; Foray et al., 2024; Zhou et al., 2024) and recalibrating movement in response to visual displacements (e.g., visuomotor rotation; Fernández-Ruiz et al., 2004; Kitago et al., 2013; Zhou et al., 2017; Bansal et al., 2019; Bindra et al., 2021).

Similar to adaptation, studies of bimanual movements have provided valuable insights into how the motor system coordinates complex behaviors. A common approach has involved tasks that analyze how rhythmic coordination between the two hands emerges under different conditions (Peper et al., 1995; Pollok et al., 2005; Serrien, 2008, 2009; Tallet et al., 2010). Furthermore, a number of studies have examined learning during bimanual movements when there are separate task goals and/or cooperative control (Diedrichsen et al., 2004; Diedrichsen, 2007; Diedrichsen and Gush, 2009; Buckingham et al., 2010; Howard et al., 2010; Yokoi et al., 2011, 2014; Kagerer, 2016; Doost et al., 2017; Schaffer and Sainburg, 2017; Bulens et al., 2018). Bimanual interference, which is commonly demonstrated by the difficulty of engaging the two upper limbs in asymmetrical tasks (i.e., rubbing one's stomach while patting the head), has also been explored to better understand bimanual coordination. For example, during a reaching task when one hand received altered visual feedback and the other only kinesthetic feedback, applying resistive forces led to interference between the two hands. The interference did not have the same effect on the unperturbed limb, suggesting that the type of feedback may influence bimanual coordination (Brunfeldt et al., 2021). In other studies, when the two hands act together (White and Diedrichsen, 2010; Reichenbach et al., 2013; Salimpour and Shadmehr, 2014), adjustments in the distributed control across the limbs were nonuniform, with one limb contributing more to the combined motor output.

As described above, the majority of bimanual upper limb studies utilize tasks that require similar functions (e.g., trajectory control, tapping). However, bimanual coordination in daily life typically necessitates that the two limbs perform different but complementary motor functions (e.g., hammering a nail—one hand stabilizes the nail while the other swings the hammer). Subsequently, there is a substantial gap in our understanding of how the two hands coordinate control in accomplishing a single task, particularly when the required motor functions are not the same. In this study, our objective was to address this gap by developing a novel bimanual coordination task that requires the two upper limbs to jointly control different aspects of a single virtual object and measured how perturbations of one feature of the coordinated task influenced the spatiotemporal properties of motions in the other unperturbed limb. Based on the aforementioned studies, we hypothesized that perturbing either motor function would lead to interference in the unperturbed limb due to subjects learning to coordinate the joint task. Furthermore, given that prior research has suggested interlimb differences due to hand dominance (Sainburg and Kalakanis, 2000; Bagesteiro and Sainburg, 2002; Schaffer and Sainburg, 2021), we hypothesized that limb dominance would contribute to differences in kinematic measures when each upper limb was perturbed during the bilateral task. Our behavioral results provide interesting insights into the adaptation of bimanual control that are not aligned with our initial hypotheses, suggesting possible differences in the underlying neural mechanisms and interhemisphere communication.

## Materials and Methods

### Participants

A total of 120 healthy participants with no known neurological impairments were recruited to participate in this research study. The study consisted of Experiment 1: Bimanual Coordination of Distinct Motor Actions and Experiment 2: Control for Motor Action Duration Differences. Human subjects for both experiments were recruited at a location which will be identified if the article is published. In Experiment 1, four participants were excluded from the data analysis due to their mismanipulation of the robotic manipulandum or knob (remaining total 76 participants: 25 male, 50 female, 1 nonbinary; aged 21.01 ± 6.49 years). Handedness of participants was measured by the Edinburgh Handedness Inventory (Oldfield, 1971). Seven participants were left-handed (Laterality Quotient <0), and 69 participants were right-handed (Laterality Quotient >0). In Experiment 2 (40 participants: 14 male, 26 female; aged 20.68 ± 3.59 years), two participants were left-handed and 38 participants were right-handed. Each participant only performed a single experimental paradigm and was naive to the task. The study protocol was approved by the University of California Davis Institutional Review Board, and all participants provided written informed consent.

### Experimental setup

**Figure 1.**
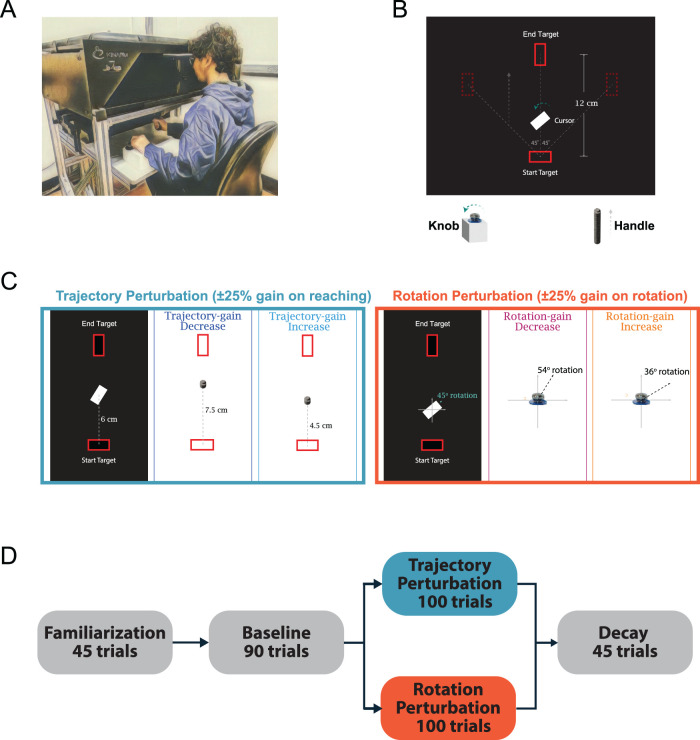
Experimental setup and paradigm. ***A***, Participants were seated in an upright position facing the robotic manipulandum. Visual feedback was provided by the mirror display obstructing forearm visibility. Participants made reaching movements using the manipulandum handle and rotation using the knob during the task. Reaching movements were made between two targets in the directions away and toward the body. ***B***, Participants were instructed to move the cursor from a rectangle starting position (2.5 cm × 1.5 cm with 0° orientation) located at the center of the screen (20 cm from the body on the sagittal axis) to a rectangle target (2.5 cm × 1.5 cm) located a set distance away (12 cm for Experiment 1 and 6 cm for Experiment 2) from the starting position at one of three possible directions (45, 90, and 135°) and match the orientation of the goal target (0 or 90°), where we refer the direction of *x*-axis as 0°. Each participant used one of the hands to hold the handle of the robot and controlled the trajectory of the cursor while the other hand rotated the knob to control its orientation. ***C***, During the training block, a gain was applied to the visual feedback of one aspect of the bimanual task (the movement trajectory or the cursor orientation). The gain was either an increase or a decrease of 25% (i.e., the visual feedback showed movement/rotation that was either 25% longer or shorter than the actual hand movement/rotation). ***D***, The experiment task started with a familiarization block (45 trials). During a familiarization trial, there was only one target located 12 cm (Experiment 1) or 6 cm (Experiment 2) away from the starting position at 90° with 0° orientation. Participants then performed a baseline block consisting of 90 trials with goal targets at one of the three random directions (45, 90, and 135°). The orientation of the targets was set to 90° (for the rest of the experiment). After the baseline phase, participants performed a block of 100 training trials. During the training block, a gain was applied to the visual feedback of one aspect of the bimanual task (the movement trajectory or the cursor orientation). At the end of the experiment, participants performed a decay block of 45 trials without visual perturbations (same as the baseline trials) to assess how the decay of learning was acquired during the training block. For a breakdown of participant numbers and demographics within each training block based on perturbation type, see Extended Data Figure 1-1.

We utilized a bimanual experimental task that required the two limbs to perform two distinct motor functions. Participants were seated in front of the bimanual robotic manipulandum (KINARM End-Point Lab; BKIN Technologies) at a height where the head could rest comfortably on the system's headrest (Fig. 1*A*). A horizontal mirror display occluded the participant's view of the forearms to limit visual feedback of the upper limbs and hand position to only what was observed on the screen. Computer-generated visual feedback from the task was projected onto the mirror from a downward-facing LCD monitor positioned directly above (1,920 × 1,080 pixel resolution and 60 Hz refresh rate). Participants were instructed to complete the bimanual task where they performed visually guided reaching movements using the handle of the robotic manipulandum, while rotating a knob (3.8 cm in diameter) connected to the KINARM (Fig. 1*B*). A solid white rectangular cursor (2 cm × 1 cm) was shown to match the handle position, and the rotation of the knob controlled the orientation of the cursor (Fig. 1*B*). During the experiments, the KINARM continuously measured handle position plus velocity and rotation of the knob at a sampling rate of 1,000 Hz while simultaneously displaying visual feedback to participants.

10.1523/ENEURO.0112-25.2025.f1-1 Figure 1-1 **Demographic information for Experiments 1 and 2.** Demographic breakdown of participants in Experiment 1: Bimanual Coordination of Distinct Motor Actions and Experiment 2: Control for Motor Action Duration Differences. Download Figure 1-1, DOCX file.

### Experimental paradigm

The goal of Experiment 1 was to investigate the integration of spatiotemporal information from both limbs performing different motor functions (reach and rotation) and subsequently determine the required relative change in the respective motor behaviors. Experiment 2 was utilized as a control experiment to investigate the effects of reaching amplitude on visual perturbations to different motor functions.

During each trial, participants in Experiment 1 were instructed to move the cursor from a rectangular central starting position (2.5 cm × 1.5 cm with 0° orientation) located at the center of the screen (20 cm from the body on the sagittal axis) to a rectangular goal target (2.5 cm × 1.5 cm) located 12 cm away from the starting position at one of three possible directions (45, 90, and 135°) and match the orientation of the target (0 or 90°; Fig. 1*B*), where we refer the direction of the *x*-axis as 0°. In Experiment 2, participants moved the cursor to a rectangular target located 6 cm away from the starting position. To prevent participants from rotating the cursor prior to movement onset, the cursor did not respond to rotation unless it was moved from the start position. Each participant used one hand to hold the handle of the robot and control the trajectory of the cursor while the other hand rotated the knob to control its orientation. We encouraged participants to make rapid and uninterrupted movements by giving them visual and auditory feedback of their movement velocity. The target was filled green and a short-duration beep tone (at a frequency of 440 Hz for 200 ms) was presented when their peak movement velocity was 25–35 cm/s for Experiment 1 and 12–22 cm/s for Experiment 2. Movements above or below the desired range were indicated with a red (too fast) or yellow (too slow) target, and no auditory feedback was given. After reaching the target, participants were required to wait at the target for 500 ms, after which the robot handle automatically brought the participant's hand back to the initial position and reset the cursor orientation to 0° before beginning the next trial. While the participant's hand was moved back, the cursor was not visible.

### Experimental tasks

Experiments 1 and 2 started with a familiarization block (45 trials). During a familiarization trial in Experiment 1, there was one target located 12 cm away from the starting position on the sagittal axis of the body with 0° (horizontal) orientation. In Experiment 2, the target was located 6 cm away from the start target. Participants completed this block to become familiar with the reaching movements and attain the desired movement velocity. No knob rotation was required in this block. Participants next performed a baseline block of 90 trials with targets at one of the three randomly assigned movement directions (45, 90, and 135°). In this block of trials, the orientation of the targets was set to 90° (and this was maintained for the rest of the experiment). Each participant was instructed to make a point-to-point reaching movement while rotating the knob to match the orientation of the target. Over the baseline blocks, there were 30 trials for each of the three target directions and the last 10% of those trials served to define the participant's baseline control of trajectory and rotation toward each of the three targets.

Following the baseline block, participants performed a block of 100 training trials. During the training block, we applied a gain to the visual feedback of one aspect of the bimanual task (either movement trajectory or the cursor orientation; Fig. 1*C*). The gain was either an increase or a decrease of 25% (i.e., the visual feedback showed movement/rotation that was either 25% longer or shorter than the actual hand movement/rotation, measured in distance/angle). The gain values were made symmetrical and chosen based on prior literature (Krakauer et al., 2000) as well as to ensure that arm reaching movements would not extend beyond the normal workspace. The learning of the two types of visual perturbations was examined during the bimanual task. At the end of the experiment, participants performed a decay block of 45 trials without the visual perturbations (the relationship between movement and the visual feedback was the same as the baseline trials) to assess the decay of any learning acquired during the training block (Fig. 1*D*).

Each participant performed the task under two configurations: right-hand controlling movement trajectory and left-hand controlling orientation and vice versa. The order of which hand controlled each aspect of movement was counterbalanced to avoid bias. This protocol allowed us to evaluate potential effects of limb dominance (i.e., dominant vs nondominant hand) on the bimanual control. Thus, there were eight conditions dependent upon our three binary variables (1) which hand (right or left), (2) controlled which aspect (trajectory or orientation) of the coordination, and (3) the feedback perturbation (gain increase or decrease) that was applied. Participants (76 for Experiment 1 and 40 for Experiment 2) were divided into four groups based on the types of visual perturbation: TD (trajectory gain decrease; *N* = 20 for Experiment 1, *N* = 10 for Experiment 2), TI (trajectory gain increase; *N* = 20 for Experiment 1, *N* = 10 for Experiment 2), RD (rotation gain decrease; *N* = 17 for Experiment 1 and *N* = 10 for Experiment 2), and RI (rotation gain increase; *N* = 19 for Experiment 1 and *N* = 10 for Experiment 2) groups. For further demographic information, see Extended Data Figure 1-1. Each group of participants only experienced one type of visual perturbation throughout the whole experiment. All participants were instructed to make smooth movements (i.e., reaching directly to the target) and match the cursor to the orientation of the target. For all experiments, the instructions were given before participants performed the experiment and were repeated before the training block.

### Movement and coordination analysis

To assess the spatiotemporal information from both limbs performing different motor functions, we computed six movement parameters (described below) and examined the relative change in these metrics during the different blocks of the bimanual task:

1. Rotation amplitude (degree): the angular distance between rotation onset and offset, where rotation onset was the time at which rotation velocity first exceeded 1°/s, and rotation offset was the time at which it fell below 1°/s after the peak of rotation velocity.
2. Reaching amplitude (cm): distance of the hand position starting at movement onset until movement offset, where the movement onset and offset were defined as the time the reaching velocity first >5 and <5 cm/s after velocity peak, respectively.
3. Peak rotation velocity (degree/s): maximum rotational velocity achieved during a trial.
4. Peak reaching velocity (cm/s): maximum reaching velocity achieved during a trial.
5. Reaching duration (ms): time interval between the movement onset and offset.
6. Rotation duration (ms): time interval between the rotation onset and offset.

To quantify the temporal coordination of the two limbs performing distinct motor functions, we first normalized the magnitudes of the cursor's trajectory and rotation velocity profiles by subtracting the mean and dividing the maximum deviation of each velocity profile to bring the normalized velocity profiles ( $\tilde{M}(t)$ and $\tilde{R}(t)$) into the same scale:

$$\tilde{M}(t)=\frac{M(t)-\mathrm{mean}(M(t))}{\max(M(t))-\min(M(t))},\;\tilde{R}(t)=\frac{R(t)-\mathrm{mean}(R(t))}{\max(R(t))-\min(R(t))}.$$

**Figure 2.**
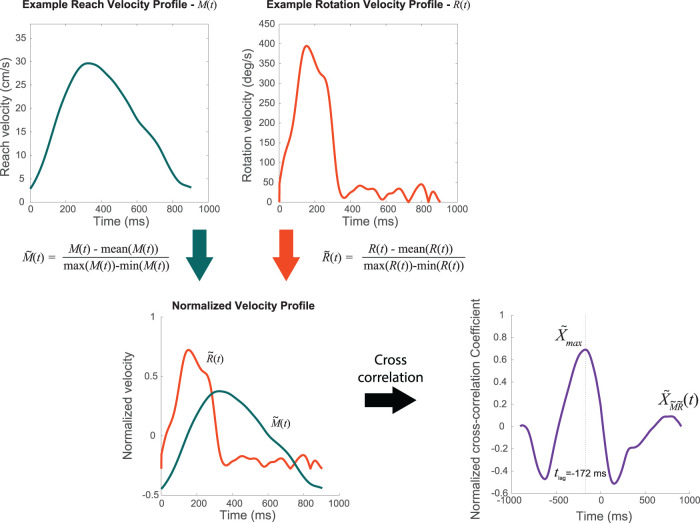
Time shift cross-correlation of normalized reaching and rotation velocity profiles. To quantify the temporal coordination of the two limbs, the reaching and rotation velocities profiles were normalized first by subtracting the mean and dividing the maximum deviation of each velocity profile. The two normalized velocity profiles ( $\tilde{M}(t)$ and $\tilde{R}(t)$, respectively) were aligned by the time the cursor left the start target and reached the goal target. Then, a cross-correlation was computed between the two normalized velocity profiles $\left(X_{\tilde{M}\tilde{R}}(t)\right)$. The normalized reaching velocity profile was used as a reference, while the normalized rotation velocity profile was shifted in time relative to the reaching profile. By varying the time shift (or lag), correlation was calculated between the two normalized profiles at each lag, indicating how well they aligned temporally. The maximum cross-correlation $\left(X_{\max}=\max(\;X_{\tilde{M}\tilde{R}}(t))\right)$ reflects the degree of similarity between the two profiles, while the corresponding lag $\left(t_{\mathrm{lag}}=\underset{t}{\max}\;(\;X_{\tilde{M}\tilde{R}}(t))\right)$ represents the time difference at which the velocity profiles were most aligned.

We then temporally aligned the normalized reaching $\left(\tilde{M}(t)\right)$ and rotation $\left(\tilde{R}(t)\right)$ velocity profiles from the time the cursor left the starting position to when it reached the target. Then, a cross-correlation was computed between the two normalized velocity profiles $\left(X_{\tilde{M}\tilde{R}}(t)\right)$. We quantified the coordination using the maximum correlation coefficient $\left(X_{\max}=\max(X_{\tilde{M}\tilde{R}}(t))\right)$ and correlation lags $\left(t_{\mathrm{lag}}=\underset{t}{\max}\;(\;X_{\tilde{M}\tilde{R}}(t))\right)$. The maximum correlation coefficient represents how well the reaching and rotation velocity profiles matched while the correlation lags represent the relative time at which the reaching velocity and rotation velocity best matched. An example of the process is plotted in Figure 2. We should note that correlation lag values are interpreted with respect to the reach movement. Therefore, positive values for correlation lags mean that the rotation followed the reaching motion, whereas negative values mean that the rotation preceded the reaching motion.

### Data collection and statistical analysis

All data were collected using KINARM End-Point Lab software and analyzed offline with MATLAB 2022a (The MathWorks) and R 4.2.1 (r-project.org). Trials that had very slow/fast movement velocity (peak velocity <0.2 or >0.6 m/s for Experiment 1 and peak velocity <0.08 or >0.35 m/s for Experiment 2; Nguyen et al., 2019) were removed (4.23% of trials for Experiment 1 and 3.95% for Experiment 2). For all statistical tests, the significance level was set to 0.05. In all cases, group data are presented as mean ± SEM. The Shapiro–Wilk test was applied to examine the normality of the data for each experimental condition. We used linear mixed effects models (LMM) in R using the *lmerTest* package (Kuznetsova et al., 2017) with the fixed effects being the participant group (TD, TI, RD, RI) and experimental block (early perturbation, late perturbation, early decay, late decay) and random effect being participants for all comparisons. The LMM models were estimated using the restricted maximum likelihood method (REML), and the significance was obtained using Kenward–Roger and Satterthwaite's approximations with *pbkrtest* package (Halekoh and Højsgaard, 2014). If significance was identified, post hoc tests were performed using the *emmeans* package and adjusted for multiple comparisons using Bonferroni–Holm corrections. Specifically, results that mention comparison to baseline were derived from a post hoc one sample *t* test comparing values against zero (baseline level of performance). Cohen's *d* was computed as effect size. All statistical analyses performed in the Results section were summarized in Extended Data Figure 3-1.

## Results

For Experiments 1 and 2, for each participant group, we compared all movement parameters, maximum correlation, and correlation lags at each experimental phase (baseline, early training, late training, early decay, and late decay phases) using a linear mixed effects model with fixed effects for movement order, limb and target direction, and a random effect for participants. We found no significant differences related to the order of performing the rotation and reaching tasks, the limbs used (dominant vs nondominant), or movements toward the three targets. Therefore, we combined the data across all conditions and focused on examining the differences among the four participant groups. In the following sections, the terms TI/RI and TD/RD refer to trajectory or rotation gain increase, and trajectory or orientation gain decrease, respectively. The results below refer to Experiment 1 unless otherwise noted. A separate section at the end describes the results from Experiment 2.

### Changes in movement parameters

#### Baseline performance

After the familiarization block, participants performed 90 baseline trials where no visual feedback perturbations were applied. The last 10% of the baseline trials served as a measurement for baseline performance as participants reached a steady state of performance on reaching and rotation. The six movement parameters (reaching duration, reaching amplitude, reaching peak velocity, rotation duration, rotation amplitude, and rotation peak velocity) were computed (the mean and SEM are summarized in Extended Data Fig. 3-2) and no significant differences were observed among the four participant groups (one-way ANOVA, *F*_(3,75)_ < 2.52, *p* > 0.05 for all movement parameters).

We observed that participants did not always reach and rotate at the same time throughout the trial. Participants only used about half of the reaching duration to rotate the cursor to the desired target orientation of 90°. That is, simultaneous coordinated movement of the two limbs occurred over about half of the total time to reach the target, even though participants were not explicitly told to coordinate their movements.

#### Performance changes during the training and decay phase

**Figure 3.**
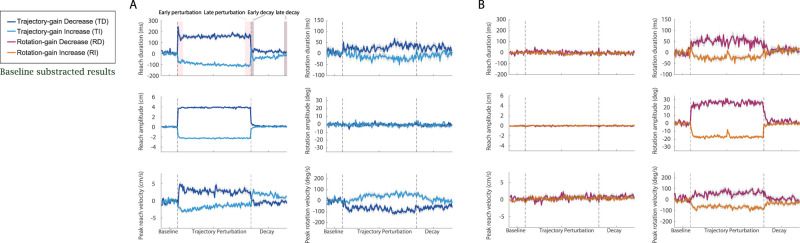
Movement parameters for Experiment 1: Bimanual Coordination of Distinct Motor Actions. ***A***, The six movement parameters (reaching amplitude, peak reaching velocity, reaching duration, rotation amplitude, peak rotation velocity, and rotation duration) throughout the whole experiment were shown for the participants in the trajectory perturbation groups. Dark blue curves were the group mean for the TD group and light blue curves were the group mean for the TI group, respectively. Light gray area showed the SEM. ***B***, The six movement parameters throughout the whole experiment were shown for the participant in the rotation perturbation groups. Dark purple curves were the group mean for the RD group and orange curves were the group mean for the RI group, respectively. Light gray area showed the SEM. All results were baseline subtracted, and statistical analyses of this data are detailed in Extended Data Figure 3-1. Raw data (mean ± SEM), is shown in Extended Data Figure 3-2.

**Figure 4.**
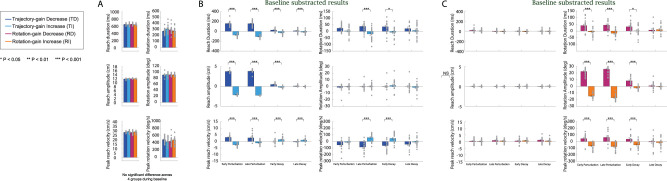
Movement parameters in different experiment phases during Experiment 1: Bimanual Coordination of Distinct Motor Actions. ***A***, The six movement parameters (reaching amplitude, peak reaching velocity, reaching duration, rotation amplitude, peak rotation velocity, and rotation duration) during baseline phase for the four participant groups [dark blue bars for the Trajectory-gain Decrease (TD) group, light blue bars for the Trajectory-gain Increase (TI) group, dark purple bars for the Rotation-gain Decrease (RD) group, and orange bars for the Rotation-gain Increase (RI) group]. ***B***, The six movement parameters for the TD and TI groups during the early perturbation, late perturbation, early decay, and late decay phases were shown for the participants in the TD and TI groups. ***C***, The six movement parameters for the RD and RI groups during the early perturbation, late perturbation, early decay, and late decay phases were shown for the participants in the RD and RI groups. All results were baseline subtracted and statistical analyses of this data are detailed in Extended Data Figure 3-1. Raw data (mean ± SEM) is shown in Extended Data Figure 3-2. The filled circles represent the individual data in each group.

After the baseline phase, perturbations were applied to the visual feedback of either the rotation or the reaching motion. We aimed to examine if the perturbation altered the reaching and rotation movements as compared with the baseline, and the extent to which these visual feedback perturbations induced changes in coordination between the two limbs. Results in the perturbation and decay phase were baseline subtracted; positive or negative changes are compared with the last 10% of the baseline levels. Data before the baseline subtraction are summarized in Extended Data Figure 3-2. As shown in Figures 3 and 4 during the training phase, participants in all groups rapidly adapted to the perturbation of visual feedback.

10.1523/ENEURO.0112-25.2025.f3-1 Figure 3-1 **Statistical analyses for Experiments 1 and 2.** Summary of the statistical analyses discussed in the Results section and performed for Experiments 1 and 2. Download Figure 3-1, DOCX file.

10.1523/ENEURO.0112-25.2025.f3-2 Figure 3-2 **Movement parameters at different experiment phases in Experiment 1.** Summary of the six movement parameters (reaching amplitude, peak reaching velocity, reaching duration, rotation amplitude, peak rotation velocity, and rotation duration) at different Experiment 1 phases (mean ± SEM) for the four participant groups. The results shown were not baseline subtracted. Download Figure 3-2, DOCX file.

##### Trajectory gain

For the trajectory perturbation groups (TD and TI), the fixed effects of group (*F*_(1,171)_ > 5.31, *p* < 0.02), experiment phase (*F*_(4,171)_ > 3.57, *p* < 0.048), and their interactions (*F*_(4,171)_ > 41.78, *p* < 0.001) were significant for the three reaching movement parameters. During the early training phase (the first 10% of perturbation trials), for the participants in the TD and TI groups, the reaching duration, reaching amplitude, and reaching peak velocity were significantly increased compared with the baseline for the TD group (*d* > 4.87, *p* < 0.001) and significantly decreased for the TI group (*d* < −2.25, *p* < 0.001). The three reaching parameters were also significantly different between the TD and TI groups (*p* < 0.001 for all cases).

Participants in both groups learned the perturbation to trajectory visual feedback early in the training phase (Fig. 3*A*,*B*), and this learning remained consistent until the late stage (the last 10% of trials, *p* > 0.53 for all cases). After sudden removal of the perturbations, during the early decay phase (the first 10% of decay trials), the reaching amplitude and duration significantly decreased for the TD group (Extended Data Fig. 3-2; *d* < −3.98, *p* < 0.001) and significantly increased for the TI group (Extended Data Fig. 3-2; *d* > 2.45, *p* < 0.001) compared with the end of the training phase. However, we still observed a significant difference between the two groups (*d* > 1.89, *p* < 0.001) and the difference in the reaching duration persisted until the end of the decay phase (*d* = 1.08, *p* = 0.0008). During the early decay phase, in response to the sudden removal of perturbations, participants in the TI group moved significantly faster (*d* = 1.68, *p* < 0.001) while participants in the TD groups moved significantly slower (*d* = −1.70, *p* < 0.001) compared with the end of the training phase. This difference in velocity between the two groups also persisted throughout the whole decay phase (*d* = −1.34, *p* < 0.001 at the end of the experimental phase).

Interestingly, the visual feedback perturbation on the reaching trajectory subsequently affected movement in the unperturbed limb controlling the object orientation for the TD and TI groups. Without perturbations on rotation, participants in both groups had a similar rotation amplitude (to match the 90° target orientation; *d* < 0.002, *p* > 0.97 for all experimental phases). However, for the TD group, from the early training phase, the rotation duration gradually increased (*d* = 0.56, *p* = 0.78) and peak rotation velocity was slightly reduced (*d* = −0.64, *p* = 0.44). A reverse relationship in these two rotation parameters was observed for the TI group (*d* = −0.097, *p* = 0.99 for the rotation duration and *d* = 0.13, *p* = 0.99 for the rotation peak velocity). As participants completed more perturbations trials, at the end of the training phase we observed a significant increase in rotation duration (*p* = 0.0095) and decrease on peak velocity (*p* = 0.0043) for the TD group compared with the baseline. The changes for the TI group were also significant (*p* = 0.048 for the rotation duration and *p* = 0.015 for the rotation peak velocity). The differences in the two parameters between the two groups were significant (*p* < 0.001 for both parameters), and these differences persisted through the early decay phase (*p* < 0.019 for both parameters) but diminished at the end of the decay phase (*p* > 0.18).

##### Rotation gain

For the rotation perturbation groups (RD and RI), the fixed effects of group (*F*_(1,159.33)_ > 6.79, *p* < 0.01), experiment phase (*F*_(4,152.45)_ > 2.44, *p* < 0.03), and their interactions (*F*_(4,152.45)_ > 4.68, *p* < 0.0014) significantly affected the three rotation parameters. Participants adapted to the visual feedback perturbation applied to the rotation rapidly; the rotation duration, amplitude, and peak velocity were significantly increased compared with the baseline for the RD group (*d* > 0.91, *p* < 0.044) and significantly decreased for the RI group (*d* < −0.96, *p* < 0.035) during early perturbation. Adaptation reached asymptote after the early training phase for both groups (comparing the three rotation parameters at the early and end stage of the perturbation phase: *p* > 0.24 for the RD group and *p* > 0.82 for the RI group). Different from the TD and TI groups, after sudden removal of the perturbations during the early decay phase, the three rotation parameters slightly decreased for the RD group (*p* > 0.35) and slightly increased for the RI group (*p* > 0.11) compared with the late training phase. The decay of the rotation adaptation was slower than the trajectory perturbation. Significant differences between the RD and RI groups were still observed for all rotation parameters during the early decay phase (*p* < 0.05). However, these differences did not persist throughout the decay phase and all parameters returned to the same baseline levels at the end of the decay phase (*p* > 0.29 for all cases).

Contrary to the results, we saw when a visual feedback perturbation was applied to the reach, perturbing the rotation affected the perturbed limb controlling the orientation, but not the unperturbed limb controlling the reaching trajectory. For the reaching parameters, no significant changes were found for reach duration, amplitude, and peak velocity for both groups at the early and late stage of the training and decay phase (*p* > 0.21 for all cases). The observed results suggested an asymmetry in the motor learning in the two limbs dependent upon which aspect of visual feedback (orientation vs trajectory) was manipulated.

### Bimanual coordination

We quantified bimanual coordination by examining the reaching and rotation velocity profiles, with the rationale that together, these two measures would quantify the extent to which reaching and rotation motions were coordinated. To summarize our findings for bimanual coordination, significant changes were observed for the movement parameters of the perturbed limbs for all four participant groups after visual perturbations were applied and after sudden removal of the perturbations during the decay phase. Visual feedback perturbations applied to the reaching trajectory subsequently affected rotation in the unperturbed limb controlling the object orientation, while this was not the case for the visual feedback perturbation applied to the rotation. In the next sections, we describe how we came to these conclusions.

#### Variations in velocity profiles

**Figure 5.**
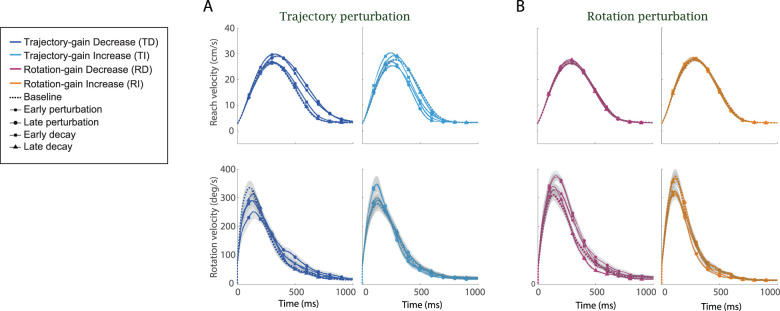
Changes in velocity profiles during Experiment 1: Bimanual Coordination of Distinct Motor Actions. ***A***, Reaching and rotation velocity profiles during baseline (dashed curves), early perturbation (curves with cross), late perturbation (curves with circle), early decay (curves with square), and late decay (curves with triangle) phases were shown for the trajectory perturbation groups (dark blue curves for the TD group and light blue curves for the TI group). ***B***, Reaching and rotation velocity profiles during the five experiment phases were shown for the rotation perturbation groups (dark purple curves for the RD group and orange curves for the RI group).

We plotted comparisons of reaching and rotation velocity profiles during the five experimental phases (baseline, early training, late training, early decay, and late decay) for the four participant groups in Figure 5*A*,*B*. The velocity profiles were plotted over a 1,000 ms window aligned at movement onset. For all groups and experimental phases, the reaching velocity profiles exhibited the same bell shape as previous studies of reaching movements (Zhou et al., 2024) while the rotation velocity profiles had a positively skewed bell shape with a long tail. In addition, participants attained peak rotation velocity sooner than attaining reaching peak velocity (Fig. 5*A*,*B*; Extended Data Fig. 6-1; time differences between the reaching and rotation velocities were quantified by the cross-correlation lags in the next subsection). These differences in the velocity profiles indicated that for the majority of the time, the rotation motion led the reaching motion during the bimanual coordination, and participants completed matching the orientation of the target before they reached the target. This explains the reason why rotation duration was always shorter than the reaching duration for all participants (Figs. 3, 4; Extended Data Fig. 3-2).

##### Trajectory gain

After visual perturbations were applied, these coordination patterns varied corresponding to the perturbation. For the TD and TI groups, compared with the baseline (dashed), the bell shape of the reaching velocity profiles (curves with the cross and circle) became wider, higher, and shifted rightward for the TD group and became narrower, lower, and shifted leftward for the TI group (Fig. 5*A*, Extended Data Fig. 6-1). The differences in the shapes of reaching and rotation velocities were quantified by the max cross-correlation in the next subsection. After the visual perturbations were removed, the reaching velocity profiles (curves with square and triangle) gradually returned to their baseline shapes (Fig. 5). For the unperturbed limb controlling the object orientation, rotation velocity became wider and lower for the TD group and became narrower and higher for the TI group. This indicates that as reaching distance increased, participants rotated slower for a longer time in the TD group and rotated faster for a shorter time in the TI group to coordinate with the perturbed limb.

##### Rotation gain

In contrast, for the RD and RI groups, the visual perturbations on rotation only affected the rotation motion, similar to the changes in reaching velocity profiles for the TD and TI groups (Fig. 5*B*). Similar to the results observed for the movement parameters, participants did not show any modulation of the unperturbed limb reaching velocities.

#### Temporal coordination

**Figure 6.**
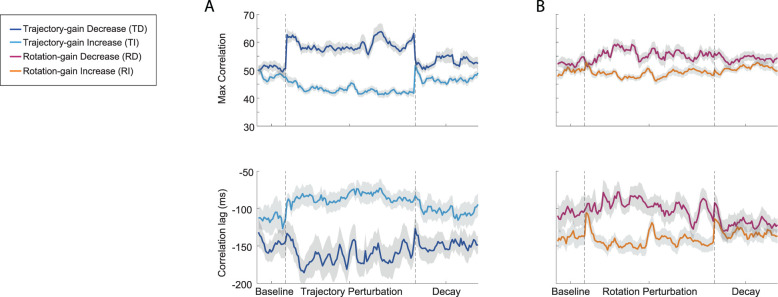
Temporal coordination throughout Experiment 1: Bimanual Coordination of Distinct Motor Actions. ***A***, Measurement of temporal interlimb coordination (maximum correlation and correlation lags) were shown for the trajectory perturbation groups. Dark blue curves were the group mean for the TD group and light blue curves were the group mean for the TI group, respectively. ***B***, Measurements of temporal interlimb coordination were shown for the rotation perturbation groups. Dark purple curves were the group mean for the RD group and orange curves were the group mean for the RI group, respectively. Light gray area showed the SEM. Statistical analyses of this data are detailed in Extended Data Figure 3-1. Raw data (mean ± SEM) is shown in Extended Data Figure 6-1.

To quantify the temporal bimanual coordination, we normalized the velocity profiles for reaching and rotation and computed the time-shifted cross-correlation (see Materials and Methods). The maximum time-shifted cross-correlation reflects the degree of similarity between the two profiles, while the corresponding lag represents the time difference at which they were most aligned (note that the negative lag values represent the time difference that the rotation velocity lead the reaching velocity, positive lag values represent the opposite). These results for the four participant groups are depicted in Figure 6*A*,*B* for the whole experiment.

10.1523/ENEURO.0112-25.2025.f6-1 Figure 6-1 **Measurements of interlimb coordination at different experiment phases in Experiment 1.** Summary of interlimb coordination measurements (max correlation and correlation lags) at different Experiment 1 phases (mean ± SEM) for the four participant groups. The results shown were not baseline subtracted. Download Figure 6-1, DOCX file.

**Figure 7.**
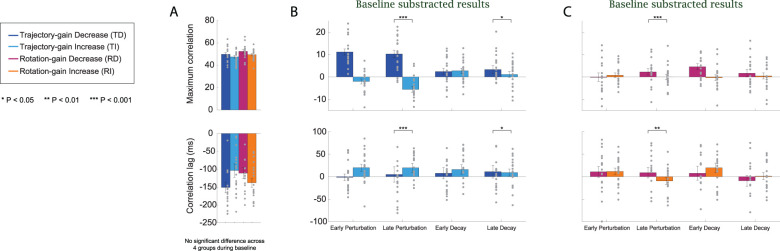
Temporal coordination at different experiment phases during Experiment 1: Bimanual Coordination of Distinct Motor Actions. ***A***, Measurements of temporal interlimb coordination (maximum correlation and correlation lags) during the baseline phase were shown for the four participant groups (dark blue bars for the TD group, light blue bars for the TI group, dark purple bars for the RD group, and orange bars for the RI group). ***B***, Changes in the temporal coordination (baseline subtracted) during early perturbation, late perturbation, early decay, and late decay phases were shown for the trajectory perturbation groups. ***C***, Changes in the temporal coordination during (baseline subtracted) early perturbation, late perturbation, early decay, and late decay phases were shown for the rotation perturbation groups. All results were baseline subtracted and statistical analyses of this data are detailed in Extended Data Figure 3-1. Raw data (mean ± SEM) is shown in Extended Data Figure 6-1. The filled circles represent the individual data in each group.

During the baseline phase, although we found that there were no significant differences in the maximum correlation (one-way ANOVA, *F*_(3,75)_ = 2.12, *p* = 0.11) and correlation lags (*F*_(3,75)_ = 2.18, *p* = 0.098) among the four participant groups, the correlation lags for the TD and RI groups were longer than the other two groups (Extended Data Fig. 6-1, Fig. 7*A*). To confirm these differences, we subtracted the maximum correlation and correlation lags from the baseline and plotted the results for different experimental phases for the four groups in Figure 7*B*,*C* (note that the positive values in the baseline subtracted correlation lags represented the time difference between rotation velocity and reaching velocity decreased compared with baseline while negative values represented the opposite).

##### Trajectory gain

As depicted in Figures 6*A* and 7*B*, during the training phase, the reaching and rotation movements correlated better for the TD group after perturbations were applied (the maximum correlation was significantly increased compared with baseline; *d* = 1.83, *p* < 0.001 for early training and *d* = −1.71, *p* < 0.001 for late training) and correlated less for the TI group (*d* = −0.29, *p* > 0.05 for early training and *d* = 0.91, *p* = 0.047 for late training). At the end of the training phase, the interlimb coordination between the two groups was significantly different (*d* = 3.00, *p* < 0.0001), and this difference persisted even after the perturbations were removed (*d* = 0.72, *p* = 0.024 for the late decay phase).

During the training phase, the correlation lags slightly decreased for the TD group and slightly increased for the TI group. The changes were not significantly different from their baseline values (*p* > 0.05). However, the leading time of the rotation over the reaching (correlation lags) for the TI group was significantly reduced compared with the time for the TD group (Extended Data Fig. 6-1; *d* = −1.24, *p* = 0.0001 for early training phase and *d* = −1.06, *p* = 0.001 for late training phase). This difference also persisted through the decay phase (*d* = −0.82, *p* = 0.011 for late decay phase).

##### Rotation gain

When visual perturbations were applied to the rotation, only small differences in the coordination were observed for the RD and RI groups which were not significant (for both maximum correlation and correlation lags, *p* > 0.05 for all cases). However, a significant difference in interlimb coordination between the two groups was observed at the end of the perturbation phase, resulting from specific changes in the rotation velocity profiles (Fig. 5; *d* = 1.18, *p* = 0.0006 for maximum correlation and *d* = 1.09, *p* = 0.0013 for correlation lags). The difference diminished after the perturbations were removed (*p* > 0.05 for all decay phases).

#### Variations in movement parameters and bimanual coordination in Experiment 2

**Figure 8.**
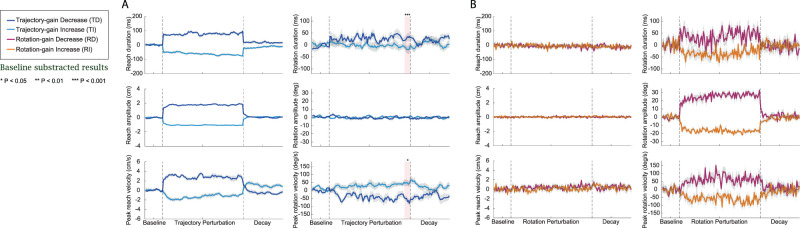
Movement parameters for Experiment 2: Control for Motor Action Duration Differences. ***A***, The six movement parameters (reaching amplitude, peak reaching velocity, reaching duration, rotation amplitude, peak rotation velocity, and rotation duration) throughout Experiment 2 are shown for the participants in the trajectory perturbation groups. Dark blue curves are the group mean for the TD group and light blue curves are the group mean for the TI group, respectively. Light gray area shows the SEM. Light pink area refers to the late perturbation phase. ***B***, The six movement parameters throughout the experiment are shown for participants in the rotation perturbation groups. Dark purple curves are the group mean for the RD group and orange curves are the group mean for the RI group, respectively. Light gray area shows the SEM. All results were baseline subtracted and statistical analyses of this data are detailed in Extended Data Figure 3-1. Raw data (mean ± SEM) is shown in Extended Data Figure 8-1.

**Figure 9.**
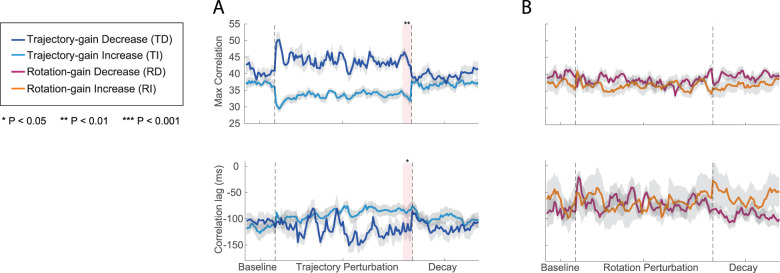
Temporal coordination throughout Experiment 2: Control for Motor Action Duration Differences. ***A***, Measurement of temporal interlimb coordination during Experiment 2 (maximum correlation and correlation lags) are shown for the trajectory perturbation groups. Dark blue curves are the group mean for the TD group and light blue curves are the group mean for the TI group, respectively. Light pink area refers to the late perturbation phase. ***B***, Measurements of temporal interlimb coordination are shown for the rotation perturbation groups. Dark purple curves are the group mean for the RD group and orange curves are the group mean for the RI group, respectively. Light gray area shows the SEM. Statistical analyses of this data are detailed in Extended Data Figure 3-1. Raw data (mean ± SEM) is shown in Extended Data Figure 9-1.

In Experiment 1, we observed that the reaching duration was approximately double that of the rotation duration across all four participant groups. To examine the potential effects of reaching amplitude and duration on the asymmetry observed during recalibration of reach and rotation movements, we conducted Experiment 2 employing the same paradigm as Experiment 1 but with the reaching target distance reduced by half, from 12 to 6 cm. Changes in movement parameters and bimanual coordination are presented in Figures 8 and 9. For clearer visualization of group differences, the curves were smoothed using a moving average with a window size of five trials; however, all statistical analyses were performed on the raw data.

10.1523/ENEURO.0112-25.2025.f8-1 Figure 8-1 **Movement parameters at different experiment phases in Experiment 2.** Summary of the six movement parameters (reaching amplitude, peak reaching velocity, reaching duration, rotation amplitude, peak rotation velocity, and rotation duration) at different Experiment 2 phases (mean ± SEM) for the four participant groups. The results shown were not baseline subtracted. Download Figure 8-1, DOCX file.

10.1523/ENEURO.0112-25.2025.f9-1 Figure 9-1 **Measurements of interlimb coordination at different experiment phases in Experiment 2.** Summary of interlimb coordination measurements (max correlation and correlation lags) at different Experiment 2 phases (mean ± SEM) for the four participant groups. The results shown were not baseline subtracted. Download Figure 9-1, DOCX file.

During the baseline phase, the reaching amplitude in each group was approximately half of that in Experiment 1 (5.69 ± 0.02 cm across four groups; Extended Data Fig. 8-1). As a result of the shorter amplitude, all participants in Experiment 2 reached the target with approximately half the velocities (14.90 ± 0.20 cm/s; Extended Data Fig. 8-1) and shorter durations (494.088 ± 4.48 ms; Extended Data Fig. 8-1) compared with those in Experiment 1, whereas the rotation parameters did not differ significantly between the two experiments (for Experiment 2, rotation amplitude: 91.078 ± 0.65°; peak rotation velocity: 444.88 ± 16.48°/s; rotation duration: 354.14 ± 10.63 ms).

In Experiment 2, during the perturbation phase, as shown in Figure 8, changes in all six movement parameters across the four perturbation groups were consistent with those observed in Experiment 1 (Fig. 3). Participants in all groups adapted to the perturbation early in the training phase, and this adaptation remained stable through the late stage. Importantly, similar to Experiment 1, perturbations applied to the reaching trajectory influenced the unperturbed limb responsible for object rotation in the TD and TI groups. Specifically, in both groups, perturbing the trajectory altered the rotation duration and peak rotation velocity. These measures were significantly different between the TD and TI groups during the late training phase (*d* = 2.05, *p* < 0.0001 for rotation duration and *d* = −1.02, *p* < 0.05 for peak rotation velocity). On the contrary, visual perturbations applied to rotation produced no significant effects on reaching parameters, as indicated by both maximum correlation and correlation lag measures (*p* > 0.05 for all cases).

For bimanual coordination, compared with the baseline in Experiment 1, the shorter reaching amplitude and slower reaching velocity in Experiment 2 led to a reduction in the maximum correlation (38.25 ± 0.83 across four groups; Extended Data Fig. 9-1) between the two movements. However, correlation lags were also reduced (−97.86 ± 9.13 ms across four groups; Extended Data Fig. 9-1), indicating improved temporal coordination between reaching and rotation. In Experiment 2, as shown in Figure 9, the overall patterns were similar to those in Experiment 1. After the trajectory perturbations were applied, the magnitude of the reaching and rotation movements (maximum correlation) became more correlated in the TD group and less correlated in the TI group (*d* = −1.68, *p* = 0.0015). The difference in the maximum correlation between the two groups was significant during the late training phase (*d* = −1.68, *p* = 0.0015). At the same time, the leading time of rotation relative to reaching (correlation lag) was significantly smaller in the TI group compared with the TD group at late training (*d* = 1.15, *p* = 0.019). These group differences did not persist into the decay phase (*p* > 0.05 for all cases). For participants in the RD and RI groups, perturbations of rotation did not significantly affect either maximum correlation or correlation lags (*p* > 0.05 for all cases).

In summary, the results of Experiment 2 confirmed that the asymmetry in motor learning and bimanual coordination depended on which aspect of visual feedback (orientation vs trajectory) was manipulated and was independent of reaching amplitude or duration.

## Discussion

In this study, we introduced a novel experimental paradigm to assess bimanual coordination where each limb enacts different but complementary motor functions. In our task, participants controlled the trajectory and orientation of a single virtual cursor by performing visually guided reaching movements with one hand using a robotic handle, while the other hand rotated a knob. We applied visual perturbations to one feature of the coordination task to understand how this may influence the spatiotemporal properties of the other, unperturbed limb. We found an asymmetry in the motor learning of our task; perturbing the reaching movement affected the rotation movement of the unperturbed limb, but the opposite was not observed. Contrary to our initial hypothesis, limb dominance did not contribute to this asymmetry. However, since our participant pool was 92% right-handed, we cannot claim this applies to left-handed individuals. Furthermore, we found that changes in cursor orientation occurred before changes in cursor trajectory in the bimanual task.

### Comparison with previous bimanual coordination studies

As described above, our study examined the coordination of two distinct, yet complementary motor functions. Many studies have investigated bimanual movement, however, a majority of them have used tasks where the two limbs perform similar functions (i.e., both arms reach) and/or a rhythmic constraint is applied to the movement (i.e., the two arms move with respect to a specific frequency ratio; Swinnen et al., 1997; Debaere et al., 2004). For a further review, see Rueda-Delgado et al. (2014). In contrast, in the current study we tasked participants with coordinating reach and rotation motions to guide a single virtual cursor to a different target location and orientation. Participants were instructed to coordinate the respective motions, with the main constraint placed on maintaining a reaching velocity within a specific range. To our knowledge, few if any studies exist similar to ours. A plethora of work has investigated bimanual coordination of a shared physical/virtual object (Woytowicz et al., 2018; Schaffer and Sainburg, 2021), such as cooperative control of a cursor where one hand controls the horizontal position and the other steers the vertical (Doost et al., 2017), or commonly where the single cursor is the average position of the two controlling hands (Diedrichsen, 2007; Diedrichsen and Dowling, 2009; Mutha and Sainburg, 2009; Kitchen et al., 2023). Our experimental task aims to better understand bimanual movements by probing how different motor functions are coordinated between the upper limbs. This is in contrast to prior bimanually asymmetric tasks that have been utilized (e.g., different perturbations, movement rhythms assigned to each limb; Diedrichsen and Dowling, 2009; Li et al., 2009; Sisti et al., 2011) which have established tractable methods for investigating bimanual coordination and provided key insights but are still vastly different from day-to-day bimanual function. In our study, we aimed to use an experimental paradigm more aligned with the daily functions of the two hands. Our goal was to better understand how fundamental movements are coupled by our upper limbs to produce synchronized, coordinated bimanual movements common in our day-to-day tasks (e.g., pouring a drink, opening a door with a key).

### Asymmetry in recalibration of bimanual coordination

Our results reveal an asymmetry when recalibrating the reach or rotation movement after the introduction of a gain perturbation. We found that a change in gain when applied to reach influences the rotation movement, but not vice versa. In addition, this asymmetry was independent of limb dominance among right-handed participants. Regardless of whether the participant's dominant hand was assigned to control reach or rotation, perturbing the reaching hand affected the rotating hand, but perturbing the rotation hand had little effect on the reaching hand.

There may be several explanations for this. One is that reach and rotation movements have inherently different spatial representations due to the differing nature of the two movements; commanding the reach enacts shoulder and elbow flexion/extension, while commanding the rotation is more localized and mostly limited to wrist and finger movements. The shoulder and elbow movements comprising a reach and grasp task have been shown to take a greater proportion of time than wrist movement (Lacquaniti and Soechting, 1982). Given this spatial and temporal dissimilarity, it is plausible that a visual feedback perturbation applied to a reaching motion—which may dominate spatially and temporally over the rotation—in turn has a more significant effect on the rotation when perturbed. To investigate this further, we conducted Experiment 2 where the reaching distance, and thus the movement duration difference between the reach and rotation motions, was substantially reduced. As we observed Figures 8 and 9, despite nearly halving the reaching velocity, we saw the same asymmetrical results as in Experiment 1. This further supported our findings that perturbing the reaching trajectory has greater influence on the unperturbed limb and is not simply a consequence of modulating reaching kinematics. This is further seen in the experimental phase when the visual feedback perturbation was removed from the reach and rotation (decay). By the end of the decay phase, all rotation parameters had returned to baseline levels. In contrast, the reaching parameters continued to exhibit differences from baseline levels. These longer-lasting after-effects of adaptation are analogous to those of visuomotor rotation and force field adaptation observed in previous research (Shadmehr and Mussa-Ivaldi, 1994; Block and Celnik, 2013; Wang and Lei, 2015). This also suggests that an internal model was formed during the training phase, perhaps more strongly for the trajectory perturbation, therefore continuing to compensate for the perturbations as an after effect when they were removed.

Research by Sainburg et al. suggests that hand dominance leads to interlimb differences, where the dominant arm and nondominant arm exhibit specialization for different functions (Sainburg and Kalakanis, 2000; Bagesteiro and Sainburg, 2002). One example is a study that, similar to our experimental goal, employed an experimental paradigm more characteristic of day-to-day tasks. Two hands were mechanically coupled, where one hand was assigned the role of stabilizing while the other hand performed a reach. Their results showed that right-handed subjects exhibited greater performance with the dominant arm reaching and the nondominant arm stabilizing (Woytowicz et al., 2018). Another more recent study has shown that perturbations during bilateral movements to the dominant arm led to differences in kinematic measures in the nondominant arm, but the opposite did not hold (Schaffer and Sainburg, 2021). Strikingly, our results showed no significant effect of limb dominance (i.e., dominant vs nondominant hand) during perturbations of the reach and rotation. Instead, we observed that the motor function, rather than the dominance of the perturbed arm, leads to asymmetric responses. These findings may in part be explained by prior research that suggests the brain selects “primary” and “secondary” roles for each hand, depending on the spatial relationship between the hand and the task goal (Johansson et al., 2006; Hughes et al., 2013). It is suggested that maintaining flexibility regarding which hand assumes the primary role in a task increases effectiveness in task completion. This is supported by another study showing that interlimb coupling (how limb movements affect one another) in a sequential bimanual reach, grasp, and rotation task was influenced by task demands and context, also suggesting that the primary task role is not always assigned to the habitual and/or dominant hand (Hughes et al., 2013). Indeed, in day-to-day bimanual coordination tasks there are many instances when the preferred and/or dominant hand may take on a supporting role while the other nondominant hand enacts the primary motion (i.e., grasp, reach, pull; Johansson et al,., 2006; Theorin and Johansson, 2010; Hughes et al., 2013). For example, when pouring a bottle of water, the preferred hand may take on the supporting role and hold the bottle while the nonpreferred hand twists the cap, so that afterward the preferred hand can complete the primary task of pouring the water (example adopted from Hughes et al,., 2013). Our results may be aligned with a similar phenomenon, where based on the motor function required for the task, each hand remains flexible to assume the primary or secondary role. Furthermore, a recent study by Kitchen et al. suggests that bimanual coordination may be greatly task dependent. The study showed that varying gain manipulation throughout the task in turn affected spatial covariation and independent control between the hands (Kitchen et al., 2023). Our findings may align with these results; in the current study, two arms act on a spectrum of independent and codependent control, possibly due to task constraints. This could also explain why we observed temporal variability of the two motions, where the rotation consistently preceded the reach (Fig. 2), and the orientation of the target was matched before the target was reached. It is possible that one motion was performed earlier in the task in order to divide feedback and feedforward responses between the two motions, allowing the remainder of the task to be focused on the other motion.

### Neural mechanisms involved in coordination of complementary motor functions

In addition to measures of behavior, prior studies have investigated bimanual coordination from a neural perspective (Swinnen, 2002 for a detailed review of neural correlates of bimanual coordination). These investigations have revealed many cortical areas (e.g., the supplementary motor area, primary motor cortex, dorsolateral premotor cortex and parietal cortex) that all play important roles in bimanual coordination (Sadato et al., 1997; Donchin et al., 1998, 2002; Wenderoth et al., 2005; Maki et al., 2008; Meister et al., 2010). Although the exact mechanisms remain unclear, there is extensive evidence that bimanual coordination involves interhemispheric brain activity (Gerloff and Andres, 2002; Swinnen, 2002; Carson, 2005). Naturally, when examining neural correlations of bimanual coordination, the question prevails whether performance of unimanual movements translates to performance of bimanual movements (i.e., practicing piano with each hand separately before together). Studies have suggested that additional resources may be necessary to incorporate both limbs’ movements into one unified action, rather than just simply summing the respective activity of unimanual movements (Steinberg et al., 2002; Rokni et al., 2003).

The asymmetric recalibration to perturbations between the reach and rotation motions that we report may suggest important implications for how complex bimanual coordination is lateralized across the brain. Particularly, it is notable that limb handedness did not appear to affect task performance, but the type of motor function that was perturbed (regardless of which limb was controlling it) was significant. This could reflect a difference in the ability to control course (object trajectory) versus fine (object orientation) features of the coordination. Based on previous work, the common belief is that the brain's left hemisphere dominates regulation of movement, specifically limb trajectory, and the right hemisphere holds other specialized functions and is responsible for limb position and posture (Sainburg, 2002; Serrien et al., 2006). As described above, the coordination of reach and rotation may exist on a spectrum of independent and codependent control, with the rotation being more codependent. As such, it could be that the coordinated task engages bilateral networks across the brain, but a greater engagement may come from the brain hemisphere controlling the trajectory, given the stronger responses we observe from perturbing the movement trajectory. Further investigation, in which the current task is coupled with neural imaging, could provide greater insights to address these questions on the neural basis of the behavioral asymmetry.

### Conclusion

In conclusion, we developed a novel experimental paradigm to assess bimanual coordination and found asymmetries in the effects of the respective motor learning. When visual feedback perturbations were applied to the reaching motion, participants recalibrated both the perturbed reaching limb and the unperturbed rotating limb. Surprisingly, we found no significance effect of hand dominance on these behavioral results. We believe that our findings support the prominent theory of task dependence in bimanual coordination, where the independence and codependence of the two upper limbs may be dictated by the motor task given to each limb.

## Synthesis

Reviewing Editor: David Schoppik, New York University Grossman School of Medicine Decisions are customarily a result of the Reviewing Editor and the peer reviewers coming together and discussing their recommendations until a consensus is reached. When revisions are invited, a fact-based synthesis statement explaining their decision and outlining what is needed to prepare a revision will be listed below. The following reviewer(s) agreed to reveal their identity: Philippe Lefevre, Nick Kitchen.

I would like to thank the authors for their consideration of our comments and the extensive modifications they made to the manuscript, particularly with respect to the additional experiment they have now included. I have a few, mostly minor, additional comments but I am otherwise satisfied with the authors modifications and responses to our concerns.

General

1) Given the effort the authors have gone to for including the control experiment, we would like to see at least a sentence in the abstract acknowledging this (particularly as this is added strength of the manuscript for the conclusions).

Results

2) We are still slightly unsure how the results of the statistics can be commented on as significant changes "relative to baseline" (see example pages/lines below) if the data is baseline adjusted and the models do not include the baseline phase as a level of the experimental block/phase factor outlined in the statistics section? Is it that this was a post-hoc one-sample t-test comparing values against zero (i.e. the baseline level of performance)? If so please include some detail of this in the statistics section.

a)Some examples on pg. 12, line 274; pg. 13, line 300; pg. 13, line 309 but others throughout results

3) Pg. 15, line 358 - the authors refer to Figure 5B in this section regarding trajectory gain but that panel regards the rotation perturbation (i.e. the following sub-section, where it should be referenced to instead).

Discussion

4) Concerning the work of Sainburg and colleagues a specific paper comes to mind of particular relevance to naturalistic bimanual tasks which may be of some relevance for the discussion points on pg 20 - notably this task (reference below) uses a bimanual task in which one hand stabilizes position while the other makes a reaching action, which was designed to simulate the action of slicing a loaf of bread. I don't think this changes the nature of the arguments put forward by the authors but may be useful to include and contrast from the task used in your study (particularly since they do report effects of hand dominance on these different components of the task).

a) Woytowicz, E. J., Westlake, K. P., Whitall, J. &Sainburg, R. L. (2018). Handedness results from complementary hemispheric dominance, not global hemispheric dominance: evidence from mechanically coupled bilateral movements. J. Neurophysiol., 120(2), 729-740. https://doi.org/10.1152/JN.00878.2017

5) Given the highly biased sample of right to left handers in the groups (Supplementary Figure 1-1) I think there should be some comment of this in the discussion as a limitation in determining the extent to which hand dominance had no influence on the findings.

a) Presumably this factor was dichotomized into two groups (left/right handed) rather than included as a continuous variable (i.e. laterality quotient) in statistical analysis, though some added detail of this (as well as the how it was determined that the order of task performance and the target locations had no effect on outcomes measures - Results paragraph 1) would be useful in the methods.

## References

[B1] Bagesteiro LB, Sainburg RL (2002) Handedness: dominant arm advantages in control of limb dynamics. J Neurophysiol 88:2408–2421. 10.1152/jn.00901.2001 12424282 PMC10709816

[B2] Bansal S, Murthy KG, Fitzgerald JJ, Schwartz BL, Joiner WM (2019) Reduced transfer of visuomotor adaptation is associated with aberrant sense of agency in schizophrenia. Neuroscience 413:108–122. 10.1016/j.neuroscience.2019.06.012 31228588 PMC6687512

[B3] Bindra G, Brower R, North R, Zhou W, Joiner WM (2021) Normal aging affects the short-term temporal stability of implicit, but not explicit, motor learning following visuomotor adaptation. eNeuro 8:ENEURO.0527-20.2021. 10.1523/ENEURO.0527-20.2021 PMC8519305 34580156

[B4] Block H, Celnik P (2013) Stimulating the cerebellum affects visuomotor adaptation by not intermanual transfer of learning. Cerebellum 12:781–793. 10.1007/s12311-013-0486-7 23625383 PMC3779504

[B5] Brunfeldt AT, Desrochers PC, Kagerer FA (2021) Bimanual interference increases with force demands and is facilitated by visuomotor adaptation. Neuroscience 463:57–69. 10.1016/j.neuroscience.2021.03.012 33737027

[B6] Buckingham G, Binsted G, Carey DP (2010) Bimanual reaching across the hemispace: which hand is yoked to which? Brain Cogn 74:341–346. 10.1016/j.bandc.2010.09.002 20933317

[B7] Bulens DC, Crevecoeur F, Thonnard J, Lefèvre P (2018) Optimal use of limb mechanics distributes control during bimanual tasks. J Neurophysiol 119:921–932. 10.1152/jn.00371.2017 29118194

[B8] Carson RG (2005) Neural pathways mediating bilateral interactions between the upper limbs. Brain Res Rev 49:641–662. 10.1016/j.brainresrev.2005.03.005 15904971

[B9] Debaere F, Wenderoth N, Sunaert S, Van Hecke P, Swinnen SP (2004) Cerebellar and premotor function in bimanual coordination: parametric neural responses to spatiotemporal complexity and cycling frequency. Neuroimage 21:1416–1427. 10.1016/j.neuroimage.2003.12.011 15050567

[B11] Diedrichsen J (2007) Optimal task-dependent changes of bimanual feedback control and adaptation. Curr Biol 17:1675–1679. 10.1016/j.cub.2007.08.051 17900901 PMC2230536

[B12] Diedrichsen J, Dowling N (2009) Bimanual coordination as task-dependent linear control policies. Hum Mov Sci 28:334–347. 10.1016/j.humov.2008.10.003 19131136

[B13] Diedrichsen J, Gush S (2009) Reversal of bimanual feedback responses with changes in task goal. J Neurophysiol 101:283–288. 10.1152/jn.90887.2008 18987120 PMC2637023

[B10] Diedrichsen J, Nambisan R, Kennerley SW, Ivry RB (2004) Independent on-line control of the two hands during bimanual reaching. Eur J Neurosci 19:1643–1652. 10.1111/j.1460-9568.2004.03242.x 15066160

[B14] Donchin O, Gribova A, Steinberg O, Bergman H, Vaadia E (1998) Primary motor cortex is involved in bimanual coordination. Nature 395:274–278. 10.1038/26220 9751054

[B15] Donchin O, Gribova A, Steinberg O, Mitz AR, Bergman H, Vaadia E (2002) Single-unit activity related to bimanual arm movements in the primary and supplementary motor cortices. J Neurophysiol 88:3498–3517. 10.1152/jn.00335.2001 12466464

[B16] Doost MY, de Xivry JJO, Bihin B, Vandermeeren Y (2017) Two processes in early bimanual motor skill learning. Front Hum Neurosci 11:618. 10.3389/fnhum.2017.00618 29326573 PMC5742346

[B17] Fernández-Ruiz J, Díaz R, Aguilar C, Hall-Haro C (2004) Decay of prism aftereffects under passive and active conditions. Brain Res Cogn Brain Res 20:92–97. 10.1016/j.cogbrainres.2004.01.007 15130593

[B18] Fitzgerald JJ, Zhou W, Chase SM, Joiner WM (2024) Dissociating the influence of limb posture and visual feedback shifts on the adaptation to novel movement dynamics. Neuroscience 549:24–41. 10.1016/j.neuroscience.2024.02.033 38484835

[B19] Foray K, Zhou W, Fitzgerald JJ, Gianferrara PG, Joiner WM (2024) Applied motor noise affects specific learning mechanisms during short-term adaptation to novel movement dynamics. eNeuro 12:ENEURO.0100-24.2024. 10.1523/ENEURO.0100-24.2024 PMC11747976 39592225

[B20] Gerloff C, Andres FG (2002) Bimanual coordination and interhemispheric interaction. Acta Psychol 110:161–186. 10.1016/S0001-6918(02)00032-X 12102104

[B21] Halekoh U, Højsgaard S (2014) A Kenward-Roger approximation and parametric bootstrap methods for tests in linear mixed models – the R package pbkrtest. J Stat Softw 59:1–32. 10.18637/jss.v059.i0926917999

[B22] Hosseini EA, Nguyen KP, Joiner WM (2017) The decay of motor adaptation to novel movement dynamics reveals an asymmetry in the stability of motion state-dependent learning. PLoS Comput Biol 13:e1005492. 10.1371/journal.pcbi.1005492 28481891 PMC5440062

[B23] Howard IS, Ingram JN, Wolpert DM (2010) Context-dependent partitioning of motor learning in bimanual movements. J Neurophysiol 104:2082–2091. 10.1152/jn.00299.2010 20685927 PMC2957447

[B24] Hughes CML, Mäueler B, Tepper H, Seegelke C (2013) Interlimb coordination during a cooperative bimanual object manipulation task. Laterality 18:693–709. 10.1080/1357650X.2012.748060 23439109

[B25] Johansson RS, Theorin A, Westling G, Andersson M, Ohki Y, Nyberg L (2006) How a lateralized brain supports symmetrical bimanual tasks. PLoS Biol 4:e158. 10.1371/journal.pbio.0040158 16669700 PMC1457013

[B26] Kagerer FA (2016) Asymmetric interference in left-handers during bimanual movements reflects switch in lateralized control characteristics. Exp Brain Res 234:1545–1553. 10.1007/s00221-016-4556-1 26821317

[B27] Kitago T, Ryan SL, Mazzoni P, Krakauer JW, Haith AM (2013) Unlearning versus savings in visuomotor adaptation: comparing effects of washout, passage of time, and removal of errors on motor memory. Front Hum Neurosci 7:307. 10.3389/fnhum.2013.00307 23874277 PMC3711055

[B28] Kitchen NM, Yuk J, Przybyla A, Scheidt RA, Sainburg RL (2023) Bilateral arm movements are coordinated via task-dependent negotiations between independent and codependent control, but not by a “coupling” control policy. J Neurophysiol 130:497–515. 10.1152/jn.00501.2022 37529832 PMC10655823

[B29] Krakauer JW, Pine ZM, Ghilardi M-F, Ghez C (2000) Learning of visuomotor transformations for vectorial planning of reaching trajectories. J Neurosci 20:8916–8924. 10.1523/JNEUROSCI.20-23-08916.2000 11102502 PMC6773094

[B30] Krakauer JW, Hadjiosif AM, Xu J, Wong AL, Haith AM (2019) Motor learning. Compr Physiol 9:613–663. 10.1002/j.2040-4603.2019.tb00069.x 30873583

[B31] Kuznetsova A, Brockhoff PB, Christensen RHB (2017) Lmertest package: tests in linear mixed effects models. J Stat Softw 82:1–26. 10.18637/jss.v082.i13

[B32] Lacquaniti F, Soechting JF (1982) Coordination of arm and wrist motion during a reaching task. J Neurosci 2:399–408. 10.1523/JNEUROSCI.02-04-00399.1982 7069463 PMC6564249

[B33] Li Y, Levin O, Forner-Cordero A, Ronsse R, Swinnen SP (2009) Coordination of complex bimanual multijoint movements under increasing cycling frequencies: the prevalence of mirror-image and translational symmetry. Acta Psychol 130:183–195. 10.1016/j.actpsy.2008.12.003 19166988

[B34] Maki Y, Wong KFK, Sugiura M, Ozaki T, Sadato N (2008) Asymmetric control mechanisms of bimanual coordination: an application of directed connectivity analysis to kinematic and functional MRI data. Neuroimage 42:1295–1304. 10.1016/j.neuroimage.2008.06.045 18674627

[B35] Meister IG, Foltys H, Gallea C, Hallett M (2010) How the brain handles temporally uncoupled bimanual movements. Cereb Cortex 20:2996–3004. 10.1093/cercor/bhq048 20356959 PMC3003589

[B36] Mutha PK, Sainburg RL (2009) Shared bimanual tasks elicit bimanual reflexes during movement. J Neurophysiol 102:3142–3155. 10.1152/jn.91335.2008 19793874 PMC2804413

[B37] Nguyen KP, Zhou W, McKenna E, Colucci-Chang K, Bray LCJ, Hosseini EA, Alhussein L, Rezazad M, Joiner WM (2019) The 24-h savings of adaptation to novel movement dynamics initially reflects the recall of previous performance. J Neurophysiol 122:933–946. 10.1152/jn.00569.2018 31291156 PMC6766742

[B38] Oldfield RC (1971) The assessment and analysis of handedness: the Edinburgh Inventory. Neuropsychologia 9:97–113. 10.1016/0028-3932(71)90067-4 5146491

[B39] Peper CE, Beek PJ, van Wieringen PCW (1995) Multifrequency coordination in bimanual tapping: asymmetrical coupling and signs of supercriticality. J Exp Psychol Hum Percept Perform 21:1117–1138. 10.1037/0096-1523.21.5.1117

[B40] Pollok B, Südmeyer M, Gross J, Schnitzler A (2005) The oscillatory network of simple repetitive bimanual movements. Brain Res Cogn Brain Res 25:300–311. 10.1016/j.cogbrainres.2005.06.004 16023333

[B41] Reichenbach A, Costello A, Zatka-Haas P, Diedrichsen J (2013) Mechanisms of responsibility assignment during redundant reaching movements. J Neurophysiol 109:2021–2028. 10.1152/jn.01052.2012 23365179 PMC3628035

[B42] Rokni U, Steinberg O, Vaadia E, Sompolinsky H (2003) Cortical representation of bimanual movements. J Neurosci 23:11577–11586. 10.1523/JNEUROSCI.23-37-11577.2003 14684860 PMC6740952

[B43] Rueda-Delgado LM, Solesio-Jofre E, Serrien DJ, Mantini D, Daffertshofer A, Swinnen SP (2014) Understanding bimanual coordination across small time scales from an electrophysiological perspective. Neurosci Biobehav Rev 47:614–635. 10.1016/j.neubiorev.2014.10.003 25445184

[B44] Sadato N, Yonekura Y, Waki A, Yamada H, Ishii Y (1997) Role of the supplementary motor area and the right premotor cortex in the coordination of bimanual finger movements. J Neurosci 17:9667–9674. 10.1523/JNEUROSCI.17-24-09667.1997 9391021 PMC6573404

[B45] Sainburg RL (2002) Evidence for a dynamic-dominance hypothesis of handedness. Exp Brain Res 142:241–258. 10.1007/s00221-001-0913-8 11807578 PMC10710695

[B46] Sainburg RL, Kalakanis D (2000) Differences in control of limb dynamics during dominant and nondominant arm reaching. J Neurophysiol 83:2661–2675. 10.1152/jn.2000.83.5.2661 10805666 PMC10709817

[B47] Salimpour Y, Shadmehr R (2014) Motor costs and the coordination of the two arms. J Neurosci 34:1806–1818. 10.1523/JNEUROSCI.3095-13.2014 24478362 PMC3905146

[B48] Schaffer JE, Sainburg RL (2017) Interlimb differences in coordination of unsupported reaching movements. Neuroscience 350:54–64. 10.1016/j.neuroscience.2017.03.025 28344068 PMC5489756

[B49] Schaffer JE, Sainburg RL (2021) Interlimb responses to perturbations of bilateral movements are asymmetric. J Mot Behav 53:217–233. 10.1080/00222895.2020.1760196 32375601 PMC8056248

[B51] Serrien DJ (2008) Coordination constraints during bimanual versus unimanual performance conditions. Neuropsychologia 46:419–425. 10.1016/j.neuropsychologia.2007.08.011 17904169

[B52] Serrien DJ (2009) Interactions between new and pre-existing dynamics in bimanual movement control. Exp Brain Res 197:269–278. 10.1007/s00221-009-1910-6 19565226 PMC3284251

[B50] Serrien DJ, Ivry RB, Swinnen SP (2006) Dynamics of hemispheric specialization and integration in the context of motor control. Nat Rev Neurosci 7:160–166. 10.1038/nrn1849 16429125

[B53] Shadmehr R, Mussa-Ivaldi FA (1994) Adaptive representation of dynamics during learning of a motor task. J Neurosci 14:3208–3224. 10.1523/JNEUROSCI.14-05-03208.1994 8182467 PMC6577492

[B54] Sing GC, Joiner WM, Nanayakkara T, Brayanov JB, Smith MA (2009) Primitives for motor adaptation reflect correlated neural tuning to position and velocity. Neuron 64:575–589. 10.1016/j.neuron.2009.10.001 19945398

[B55] Sisti HM, Geurts M, Clerckx R, Gooijers J, Coxon JP, Heitger MH, Caeyenberghs K, Beets IAM, Serbruyns L, Swinnen SP (2011) Testing multiple coordination constraints with a novel bimanual visuomotor task. PLoS One 6:e23619. 10.1371/journal.pone.0023619 21858185 PMC3157395

[B56] Steinberg O, Donchin O, Gribova A, De Oliveira SC, Bergman H, Vaadia E (2002) Neuronal populations in primary motor cortex encode bimanual arm movements. Eur J Neurosci 15:1371–1380. 10.1046/j.1460-9568.2002.01968.x 11994131

[B58] Swinnen SP (2002) Intermanual coordination: from behavioural principles to neural-network interactions. Nat Rev Neurosci 3:348–359. 10.1038/nrn807 11988774

[B57] Swinnen SP, Dounskaia N, Walter CB, Serrien DJ (1997) Preferred and induced coordination modes during the acquisition of bimanual movements with a 2:1 frequency ratio. J Exp Psychol Hum Percept Perform 23:1087–1110. 10.1037/0096-1523.23.4.1087

[B59] Tallet J, Barral J, James C, Hauert C-A (2010) Stability-dependent behavioural and electro-cortical reorganizations during intentional switching between bimanual tapping modes. Neurosci Lett 483:118–122. 10.1016/j.neulet.2010.07.074 20678541

[B60] Theorin A, Johansson RS (2010) Selection of prime actor in humans during bimanual object manipulation. J Neurosci 30:10448–10459. 10.1523/JNEUROSCI.1624-10.2010 20685987 PMC6634672

[B61] Wang J, Lei Y (2015) Direct-effects and after-effects of visuomotor adaptation with one arm on subsequent performance with the other arm. J Neurophysiol 114:468–473. 10.1152/jn.00298.2015 26019313 PMC4509398

[B62] Wenderoth N, Debaere F, Sunaert S, Swinnen SP (2005) Spatial interference during bimanual coordination: differential brain networks associated with control of movement amplitude and direction. Hum Brain Mapp 26:286–300. 10.1002/hbm.20151 15965999 PMC6871760

[B63] White O, Diedrichsen J (2010) Responsibility assignment in redundant systems. Curr Biol 20:1290–1295. 10.1016/j.cub.2010.05.069 20598886

[B64] Woytowicz EJ, Westlake KP, Whitall J, Sainburg RL (2018) Handedness results from complementary hemispheric dominance, not global hemispheric dominance: evidence from mechanically coupled bilateral movements. J Neurophysiol 120:729–740. 10.1152/jn.00878.2017 29742023 PMC7132323

[B65] Wu HG, Miyamoto YR, Gonzalez Castro LN, Ölveczky BP, Smith MA (2014) Temporal structure of motor variability is dynamically regulated and predicts motor learning ability. Nat Neurosci 17:312–321. 10.1038/nn.3616 24413700 PMC4442489

[B66] Yokoi A, Hirashima M, Nozaki D (2011) Gain field encoding of the kinematics of both arms in the internal model enables flexible bimanual action. J Neurosci 31:17058–17068. 10.1523/JNEUROSCI.2982-11.2011 22114275 PMC6623869

[B67] Yokoi A, Hirashima M, Nozaki D (2014) Lateralized sensitivity of motor memories to the kinematics of the opposite arm reveals functional specialization during bimanual actions. J Neurosci 34:9141–9151. 10.1523/JNEUROSCI.2694-13.2014 24990934 PMC6608246

[B68] Yousif N, Diedrichsen J (2012) Structural learning in feedforward and feedback control. J Neurophysiol 108:2373–2382. 10.1152/jn.00315.2012 22896725 PMC3545174

[B69] Zhou W, Fitzgerald J, Colucci-Chang K, Murthy KG, Joiner WM (2017) The temporal stability of visuomotor adaptation generalization. J Neurophysiol 118:2435–2447. 10.1152/jn.00822.2016 28768744 PMC5646197

[B70] Zhou W, Monsen E, Fernandez KD, Haly K, Kruse EA, Joiner WM (2024) Motion state-dependent motor learning based on explicit visual feedback has limited spatiotemporal properties compared with adaptation to physical perturbations. J Neurophysiol 131:278–293. 10.1152/jn.00198.2023 38166455 PMC11286305

