# Erratum: Kanapskyte and Garcia Arango et al., “Different But Complementary Motor Functions Reveal an Asymmetric Recalibration of Upper Limb Bimanual Coordination”

**DOI:** 10.1523/ENEURO.0162-26.2026

**Published:** 2026-06-03

In the article “Different But Complementary Motor Functions Reveal an Asymmetric Recalibration of Upper Limb Bimanual Coordination,” by Ada Kanapskyte, Jesus Alejandro Garcia Arango, Sanjay Joshi, Stephen K. Robinson, Jonathon S. Schofield, Lee M. Miller, Wilsaan M. Joiner, and Weiwei Zhou, which was published online on December 11, 2025, in the Materials and Methods, under the *Movement and coordination analysis* section, the description of rotation amplitude appeared incorrectly. The threshold for rotation onset and offset was listed as 1°/s but should be 50°/s in both cases. The authors note that 1°/s reflected an earlier threshold used during their initial analysis, whereas all final analyses were conducted using the correct threshold of 50°/s.

The first parameter should read: “Rotation amplitude (degree): the angular distance between rotation onset and offset, where rotation onset was the time at which rotation velocity first exceeded 50°/s, and rotation offset was the time at which it fell below 50°/s after the peak of rotation velocity.”

These changes do not affect the conclusion of the paper. The authors regret the error, and the online version has been corrected.

