# Supplementary material for: Different But Complementary Motor Functions Reveal an Asymmetric Recalibration of Upper Limb Bimanual Coordination
Source: eNeuro. 2026 Jan 2;13(1):ENEURO.0112-25.2025. doi: 10.1523/ENEURO.0112-25.2025 (PMC12794948; doi:10.1523/ENEURO.0112-25.2025)
Supplement: Figure 1-1 — Demographic information for Experiments 1 and 2. Demographic breakdown of participants in Experiment 1: Bimanual Coordination of Distinct Motor Actions and Experiment 2: Control for Motor Action Duration Differences. Download Figure 1-1, DOCX file. [file eneuro-13-ENEURO.0112-25.2025-s002.docx]

**Figure 1-1. Demographic information for Experiments 1 and 2.**

|  | Trajectory Decrease | | | Trajectory Increase | | | Rotation Decrease | | | | Rotation Increase | | | |
| --- | --- | --- | --- | --- | --- | --- | --- | --- | --- | --- | --- | --- | --- | --- |
| Subjects | Exp 1 | Exp 2 | | Exp 1 | | Exp 2 | Exp 1 | | Exp 2 | | Exp 1 | | | Exp 2 |
| Age | | | | | | | | | | | | | | |
| Mean | 19.90 | 19.80 | | 23.50 | | 19.90 | 20.06 | | 21.40 | | 20.42 | | | 21.60 |
| SD | 1.33 | 1.48 | | 12.24 | | 1.29 | 1.52 | | 3.27 | | 2.04 | | | 6.19 |
| Range | 18-23 | 18-22 | | 19-75 | | 19-22 | 18-23 | | 18-28 | | 18-25 | | | 18-39 |
| Number | | |  | |  | | |  | |  | |  |  | |
| Total  Number | 20 | 10 | | 20 | | 10 | 17 | | 10 | | 19 | | | 10 |
| Female | 13 | 7 | | 14 | | 7 | 10 | | 7 | | 13 | | | 5 |
| Male | 7 | 3 | | 6 | | 3 | 7 | | 3 | | 5 | | | 5 |
| Non-  Binary | 0 | 0 | | 0 | | 0 | 0 | | 0 | | 1 | | | 0 |
| Right-  Handed | 19 | 9 | | 15 | | 10 | 16 | | 9 | | 19 | | | 10 |
| Left-  Handed | 1 | 1 | | 5 | | 0 | 1 | | 1 | | 0 | | | 0 |
