# Supplementary material for: Different But Complementary Motor Functions Reveal an Asymmetric Recalibration of Upper Limb Bimanual Coordination
Source: eNeuro. 2026 Jan 2;13(1):ENEURO.0112-25.2025. doi: 10.1523/ENEURO.0112-25.2025 (PMC12794948; doi:10.1523/ENEURO.0112-25.2025)
Supplement: Figure 3-1 — Statistical analyses for Experiments 1 and 2. Summary of the statistical analyses discussed in the Results section and performed for Experiments 1 and 2. Download Figure 3-1, DOCX file. [file eneuro-13-ENEURO.0112-25.2025-s004.docx]

**Figure 3-1. Statistical analyses for Experiments 1 and 2.**

| Line # | Data structure | Type of test | 95% Confidence Interval (CI) | | | | | | | | | | | | | | | | |
| --- | --- | --- | --- | --- | --- | --- | --- | --- | --- | --- | --- | --- | --- | --- | --- | --- | --- | --- | --- |
| 252 | Normal  (Shapiro-Wilk test) | One-way ANOVA | Movement parameters during baseline in Experiment 1: | | | | | | | | | | | | | | | | |
|  |  |  | Movement parameters | | Group | | | | | | | Baseline | | | | | | | |
|  |  |  | Reaching amplitude (cm) | | TD | | | | | | | [11.6, 11.81] | | | | | | | |
|  |  |  |  |  | TI | | | | | | | [11.78, 11.92] | | | | | | | |
|  |  |  |  |  | RD | | | | | | | [11.69, 11.88] | | | | | | | |
|  |  |  |  |  | RI | | | | | | | [11.74, 11.86] | | | | | | | |
|  |  |  | Peak reaching velocity (cm/s) | | TD | | | | | | | [27.38, 28.31] | | | | | | | |
|  |  |  |  |  | TI | | | | | | | [28.04, 29.30] | | | | | | | |
|  |  |  |  |  | RD | | | | | | | [26.52, 28.48] | | | | | | | |
|  |  |  |  |  | RI | | | | | | | [27.68, 28.81] | | | | | | | |
|  |  |  | Reaching duration (ms) | | TD | | | | | | | [629.26, 653.38] | | | | | | | |
|  |  |  |  |  | TI | | | | | | | [616.71, 645.70] | | | | | | | |
|  |  |  |  |  | RD | | | | | | | [630.63, 660.36] | | | | | | | |
|  |  |  |  |  | RI | | | | | | | [619.03, 644.13] | | | | | | | |
|  |  |  | Rotation amplitude (deg) | | TD | | | | | | | [88.76, 93.66] | | | | | | | |
|  |  |  |  |  | TI | | | | | | | [89.76, 94.64] | | | | | | | |
|  |  |  |  |  | RD | | | | | | | [90.2, 93.68] | | | | | | | |
|  |  |  |  |  | RI | | | | | | | [89.99, 93.31] | | | | | | | |
|  |  |  | Peak rotation velocity (deg/s) | | TD | | | | | | | [451.49, 535.44] | | | | | | | |
|  |  |  |  |  | TI | | | | | | | [387.13, 486.77] | | | | | | | |
|  |  |  |  |  | RD | | | | | | | [381.56, 508.12] | | | | | | | |
|  |  |  |  |  | RI | | | | | | | [433.8, 535.22] | | | | | | | |
|  |  |  | Rotation duration (ms) | | TD | | | | | | | [252.79, 294.53] | | | | | | | |
|  |  |  |  |  | TI | | | | | | | [280.46, 341.62] | | | | | | | |
|  |  |  |  |  | RD | | | | | | | [261.39, 326.79] | | | | | | | |
|  |  |  |  |  | RI | | | | | | | [248.46, 301.03] | | | | | | | |
| 269-290 | Normal | Linear mixed effects model:  Reaching parameters ~ group × experiment phase + (1\|subject) | Reaching parameters during training and decay phase (baseline subtracted) in Experiment 1: | | | | | | | | | | | | | | | | |
|  |  |  | Reaching parameters | Group | Early perturb | | | Late perturb | | | | | Early decay | | | | Late decay | | |
|  |  |  | Reaching amplitude (cm) | TD | [3.66, 3.91] | | | [3.67, 4.03] | | | | | [0.37, 0.66] | | | | [0.02, 0.19] | | |
|  |  |  |  | TI | [-2.22, -2.06] | | | [-2.29, -2.19] | | | | | [-0.32, -0.11] | | | | [-0.08, 0.08] | | |
|  |  |  | Peak reaching velocity (cm/s) | TD | [2.49, 3.80] | | | [1.49, 4.04] | | | | | [-1.1, 1.02] | | | | [-1.09, 0.36] | | |
|  |  |  |  | TI | [-3.15, -1.93] | | | [-2, -0.34] | | | | | [0.63, 2.26] | | | | [0.39, 1.68] | | |
|  |  |  | Reaching duration (ms) | TD | [136.65, 181.97] | | | [132.68, 181.86] | | | | | [14.13, 43.01] | | | | [-6.37, 30.66] | | |
|  |  |  |  | TI | [-87.54, -59.70] | | | [-119.22, -93.13] | | | | | [-43.9, -9.26] | | | | [-29.4, 0.43] | | |
| 306-322 | Normal | Linear mixed effects model  Rotation parameters ~ group × experimental phase + (1\|subject) | Rotation parameters during training and decay phase (baseline subtracted) in Experiment 1: | | | | | | | | | | | | | | | | |
|  |  |  | Rotation parameters | Group | Early perturb | | | Late perturb | | | | | Early decay | | | | Late decay | | |
|  |  |  | Rotation amplitude  (deg) | RD | [19.13, 25.8] | | | [22.58, 29.26] | | | | | [5.7, 11.15] | | | | [-1.93, 3.80] | | |
|  |  |  |  | RI | [-16.68, -13.06] | | | [-19.33, -15.2] | | | | | [-3.83, -1.10] | | | | [-1.9, 0.72] | | |
|  |  |  | Peak rotation velocity (deg/s) | RD | [7.33, 80.99] | | | [20.73, 104.56] | | | | | [-4.51, 79.87] | | | | [-51.34, 37.58] | | |
|  |  |  |  | RI | [-94.96, -42.49] | | | [-105.58, -45.08] | | | | | [-84.44, -19.10] | | | | [-50.15, 16.42] | | |
|  |  |  | Rotation duration  (ms) | RD | [18.08, 63.55] | | | [25.32, 62.32] | | | | | [12.88, 51.1[ | | | | [-10.64, 24.98] | | |
|  |  |  |  | RI | [-23.22, 10.46] | | | [-30.64, -3.7] | | | | | [-18.69, 20.30] | | | | [-4.39, 26.77] | | |
| 293-305 | Normal | Linear mixed effects model  Rotation parameters ~ group × experimental phase + (1\|subject) | Rotation parameters during training and decay phase (baseline subtracted) in Experiment 1: | | | | | | | | | | | | | | | | |
|  |  |  | Rotation parameters | Group | Early perturb | | | Late perturb | | | | | Early decay | | | | Late decay | | |
|  |  |  | Rotation amplitude  (deg) | TD | [-3.74, 0.96] | | | [-2.26, 1.87] | | | | | [-3.47, 2.98] | | | | [-2.81, 3.08] | | |
|  |  |  |  | TI | [-2.43, 1.71] | | | [-1.85, 2.41] | | | | | [-2.33, 5.45] | | | | [-4.83, 1.45] | | |
|  |  |  | Peak rotation velocity (deg/s) | TD | [-87.94, -19.94] | | | [-119.83, -49.57] | | | | | [-97.01, -30.36] | | | | [-81.92, -3.76] | | |
|  |  |  |  | TI | [-32.97, 8.41] | | | [29.77, 88.30] | | | | | [13.83, 80.17] | | | | [-41.73, 19.84] | | |
|  |  |  | Rotation duration (ms) | TD | [11.46, 37.72] | | | [16.16, 60.10] | | | | | [10.50, 60.23] | | | | [-4.18, 46.67] | | |
|  |  |  |  | TI | [-12.04, 19.90] | | | [-40.48, 1.77] | | | | | [-26.68, 12.28] | | | | [-15.57, 30.06] | | |
| 325-327 | Normal | Linear mixed effects model  Reaching parameters ~ group × experimental phase + (1\|subject) | Reaching parameters during training and decay phase (baseline subtracted) in Experiment 1: | | | | | | | | | | | | | | | | |
|  |  |  | Reaching parameters | Group | Early perturb | | | Late perturb | | | | | Early decay | | | | Late decay | | |
|  |  |  | Reaching amplitude (cm) | RD | [-0.1, 0.11] | | | [-0.1, 0.08] | | | | | [-0.2, 0.09] | | | | [-0.16, 0.05] | | |
|  |  |  |  | RI | [-0.07, 0.08] | | | [-0.05, 0.08] | | | | | [-0.06, 0.14] | | | | [-0.04, 0.12] | | |
|  |  |  | Peak reaching velocity (cm/s) | RD | [-0.57, 1.27] | | | [0, 1.73] | | | | | [-0.59, 1.53] | | | | [0.01, 2.15] | | |
|  |  |  |  | RI | [-0.51, 0.81] | | | [-0.18, 1.14] | | | | | [0.29, 1.21] | | | | [-0.29, 1.28] | | |
|  |  |  | Reaching duration  (ms) | RD | [-6.88, 32.39] | | | [-21.91, 5.96] | | | | | [-25.33, 17.77] | | | | [-19.37, 13.64] | | |
|  |  |  |  | RI | [-13.4, 14.47] | | | [-19.88, 0.13] | | | | | [-20.23, -3.1] | | | | [-19.55, 9.01] | | |
| 382-383 | Normal | One-way ANOVA | Bimanual coordination during baseline in Experiment 1: | | | | | | | | | | | | | | | | |
|  |  |  | Coordination | Group | | | | | | | | Baseline | | | | | | | |
|  |  |  | Max correlation | TD | | | | | | | | [46.63, 52.98] | | | | | | | |
|  |  |  |  | TI | | | | | | | | [44.88, 50.08] | | | | | | | |
|  |  |  |  | RD | | | | | | | | [49.21, 55.52] | | | | | | | |
|  |  |  |  | RI | | | | | | | | [47.12, 51.86] | | | | | | | |
|  |  |  | Correlation Lags  (ms) | TD | | | | | | | | [-181.37, -120.17] | | | | | | | |
|  |  |  |  | TI | | | | | | | | [-141.27, -65.59] | | | | | | | |
|  |  |  |  | RD | | | | | | | | [-143.95, -77.62] | | | | | | | |
|  |  |  |  | RI | | | | | | | | [-162.61, -113.04] | | | | | | | |
| 390-397 | Normal | Linear mixed effects model  Max correlation ~ group × experimental phase + (1\|subject) | Max correlation during training and decay phase (baseline subtracted) in Experiment 1: | | | | | | | | | | | | | | | | |
|  |  |  |  | Group | | Early perturb | | | | Late perturb | | | | | Early decay | | | Late decay | |
|  |  |  | Max correlation | TD | | [8.37, 13.91] | | | | [6.76, 13.77 | | | | | [-0.45, 5.31] | | | [-0.13, 6.76] | |
|  |  |  |  | TI | | [-4.27, 0.29] | | | | [-8.21, -2.92] | | | | | [0.34, 5.21] | | | [-1.62, 3.85] | |
| 398-400 | Normal | Linear mixed effects model  Correlation lags ~ group × experimental phase + (1\|subject) | Correlation lags during training and decay phase (baseline subtracted) in Experiment 1: | | | | | | | | | | | | | | | | |
|  |  |  |  | Group | | | Early perturb | | | | Late perturb | | | | | Early decay | | | Late decay |
|  |  |  | Correlation lags (ms) | TD | | | [-16.67, 13.69] | | | | [-29.81, 39.52] | | | | | [-18.04, 33.19] | | | [-17.94, 40.28] |
|  |  |  |  | TI | | | [4.13, 35.77] | | | | [-1.23, 40.77] | | | | | [-5.61, 37.78] | | | [-6.62, 24.96] |
| 400-403 | Normal | Linear mixed effects model  Max correlation ~ group × experimental phase + (1\|subject) | Max correlation during training and decay phase (baseline subtracted) in Experiment 1: | | | | | | | | | | | | | | | | |
|  |  |  |  | Group | Early perturb | | | | Late perturb | | | | | Early decay | | | Late decay | | |
|  |  |  | Max correlation | RD | [-4.02, 3.70] | | | | [-0.75, 5.26] | | | | | [1.82, 7.29] | | | [-1.78, 5.05] | | |
|  |  |  |  | RI | [-1.39, 2.94] | | | | -2.55, 2.52 | | | | | [-3.07, 2.39] | | | [-2.35, 3.27] | | |
| 404-410 | Normal | Linear mixed effects model  Correlation lags ~ group × experimental phase + (1\|subject) | Correlation lags during training and decay phase (baseline subtracted) in Experiment 1: | | | | | | | | | | | | | | | | |
|  |  |  |  | Group | Early perturb | | | | Late perturb | | | | | Early decay | | | Late decay | | |
|  |  |  | Correlation lags (ms) | RD | [-13.6, 35.28] | | | | [-15.08, 32.91] | | | | | [-24.31, 39.46] | | | [-34.37, 16.03] | | |
|  |  |  |  | RI | [-3.67, 27.01] | | | | [-24.87, 5.73] | | | | | [-1.12, 40.89] | | | [-14.92, 17.13] | | |
| 436-439 | Normal | One-way ANOVA | Rotation parameters during late training in Experiment 2: | | | | | | | | | | | | | | | | |
|  |  |  | Coordination | Group | | | | | | | | Late training | | | | | | | |
|  |  |  | Rotation duration (ms) | TD | | | | | | | | [301.23, 437.38] | | | | | | | |
|  |  |  |  | TI | | | | | | | | [291.05, 355.94] | | | | | | | |
|  |  |  |  | RD | | | | | | | | [376.13, 474.50] | | | | | | | |
|  |  |  |  | RI | | | | | | | | [280.47, 401.68] | | | | | | | |
|  |  |  | Peak rotation velocity (deg/s) | TD | | | | | | | | [327.48, 508.73] | | | | | | | |
|  |  |  |  | TI | | | | | | | | [423.84, 527.29] | | | | | | | |
|  |  |  |  | RD | | | | | | | | [425.27, 553.34] | | | | | | | |
|  |  |  |  | RI | | | | | | | | [289.77, 512.31] | | | | | | | |
| 447-454 | Normal | One-way ANOVA | Bimanual coordination during late training in Experiment 2: | | | | | | | | | | | | | | | | |
|  |  |  | Coordination | Group | | | | | | | | Late training | | | | | | | |
|  |  |  | Max correlation | TD | | | | | | | | [41.38, 46.88] | | | | | | | |
|  |  |  |  | TI | | | | | | | | [31.34, 35.95] | | | | | | | |
|  |  |  |  | RD | | | | | | | | [36.59, 42.14] | | | | | | | |
|  |  |  |  | RI | | | | | | | | [33.99, 40.34] | | | | | | | |
|  |  |  | Correlation Lags  (ms) | TD | | | | | | | | [-163.99, -79.32] | | | | | | | |
|  |  |  |  | TI | | | | | | | | [-102.76, -61.11] | | | | | | | |
|  |  |  |  | RD | | | | | | | | [-97.46, -47.78] | | | | | | | |
|  |  |  |  | RI | | | | | | | | [-125.10,12.05] | | | | | | | |
