# Supplementary material for: Different But Complementary Motor Functions Reveal an Asymmetric Recalibration of Upper Limb Bimanual Coordination
Source: eNeuro. 2026 Jan 2;13(1):ENEURO.0112-25.2025. doi: 10.1523/ENEURO.0112-25.2025 (PMC12794948; doi:10.1523/ENEURO.0112-25.2025)
Supplement: Figure 6-1 — Measurements of interlimb coordination at different experiment phases in Experiment 1. Summary of interlimb coordination measurements (max correlation and correlation lags) at different Experiment 1 phases (mean ± SEM) for the four participant groups. The results shown were not baseline subtracted. Download Figure 6-1, DOCX file. [file eneuro-13-ENEURO.0112-25.2025-s005.docx]

**Figure 6-1**. **Measurements of interlimb coordination at different experiment phases in Experiment 1.**

| **Coordination** | **Group** | **Experiment Phase (Mean** **± SEM)** | | | | |
| --- | --- | --- | --- | --- | --- | --- |
|  |  | Baseline | Early perturb | Late perturb | Early decay | Late decay |
| Max correlation | TD | 49.80±6.78 | 60.84±6.20 | 60.12±7.73 | 52.13±8.10 | 52.67±9.02 |
|  | TI | 47.48±5.55 | 45.73±6.48 | 42.00±4.71 | 49.87±5.99 | 48.32±5.54 |
|  | RD | 52.37±6.13 | 52.81±5.46 | 55.26±7.54 | 56.15±6.28 | 53.79±6.52 |
|  | RI | 49.49±4.92 | 49.91±4.91 | 49.14±5.07 | 49.13±6.27 | 50.16±5.29 |
| Correlation  Lags  (ms) | TD | -150.77±65.38 | -150.49±72.57 | -143.91±88.41 | -141.10±70.99 | -143.17±54.24 |
|  | TI | -103.42±80.85 | -82.32±61.01 | -85.53±52.28 | -87.78±47.81 | -98.20±64.56 |
|  | RD | -110.78±64.51 | -94.10±62.96 | -103.04±52.81 | -104.47±49.10 | -120.32±35.23 |
|  | RI | -137.83±51.43 | -126.41±44.87 | -147.93±38.29 | -117.59±51.67 | -135.75±37.39 |
