# Supplementary material for: Different But Complementary Motor Functions Reveal an Asymmetric Recalibration of Upper Limb Bimanual Coordination
Source: eNeuro. 2026 Jan 2;13(1):ENEURO.0112-25.2025. doi: 10.1523/ENEURO.0112-25.2025 (PMC12794948; doi:10.1523/ENEURO.0112-25.2025)
Supplement: Figure 8-1 — Movement parameters at different experiment phases in Experiment 2. Summary of the six movement parameters (reaching amplitude, peak reaching velocity, reaching duration, rotation amplitude, peak rotation velocity, and rotation duration) at different Experiment 2 phases (mean ± SEM) for the four participant groups. The results shown were not baseline subtracted. Download Figure 8-1, DOCX file. [file eneuro-13-ENEURO.0112-25.2025-s006.docx]

**Figure 8-1. Movement parameters at different experiment phases in Experiment 2.**

| **Movement Parameters** | **Group** | **Experiment Phase (Mean ± SEM)** | | | | |
| --- | --- | --- | --- | --- | --- | --- |
|  |  | Baseline | Early perturb | Late perturb | Early decay | Late decay |
| Reaching amplitude  (cm) | TD | 5.73±0.13 | 7.30±0.16 | 7.54±0.32 | 6.14±0.24 | 5.79±0.19 |
|  | TI | 5.67±0.057 | 4.72±0.09 | 4.57±0.046 | 5.54±0.21 | 5.75±0.15 |
|  | RD | 5.69±0.17 | 5.67±0.14 | 5.70±0.09 | 5.68±0.19 | 5.74±0.11 |
|  | RI | 5.65±0.15 | 5.66±0.12 | 5.70±0.10 | 5.78±0.15 | 5.67±0.13 |
| Peak  Reaching velocity  (cm/s) | TD | 14.97±0.83 | 17.74±1.57 | 17.94±1.33 | 15.78±1.50 | 14.57±1.48 |
|  | TI | 15.05±0.82 | 13.89±0.62 | 14.05±0.74 | 16.03±0.76 | 16.01±0.88 |
|  | RD | 14.70±1.86 | 14.99±2.01 | 15.33±1.88 | 15.64±2.14 | 15.14±1.02 |
|  | RI | 14.86±1.25 | 14.88±1.59 | 15.06±1.85 | 15.63±1.67 | 15.28±1.09 |
| Reaching duration  (ms) | TD | 496.32±21.42 | 567.82±25.55 | 573.60±24.54 | 512.84±19.16 | 512.30±26.87 |
|  | TI | 479.99±14.65 | 429.59±14.44 | 408.12±12.59 | 457.23±21.10 | 469.22±17.26 |
|  | RD | 499.88±39.02 | 496.84±36.06 | 489.20±32.23 | 478.74±29.21 | 487.43±22.13 |
|  | RI | 500.16±29.80 | 496.01±34.84 | 495.57±40.10 | 493.53±36.95 | 486.12±30.00 |
| Rotation amplitude  (deg) | TD | 90.86±1.86 | 93.04±3.74 | 91.04±2.52 | 89.74±2.63 | 91.26±3.79 |
|  | TI | 90.08±2.77 | 89.64±3.53 | 90.88±2.74 | 91.09±2.49 | 90.13±3.14 |
|  | RD | 92.38±5.56 | 111.11±8.63 | 119.42±4.33 | 97.86±4.71 | 92.43±3.03 |
|  | RI | 90.99±5.21 | 77.68±4.54 | 74.20±5.63 | 85.42±6.68 | 90.59±6.40 |
| Peak rotation velocity  (deg/s) | TD | 464.36±112.93 | 460.80±113.65 | 418.10±126.68 | 405.98±107.85 | 420.89±117.11 |
|  | TI | 428.76±59.99 | 442.03±65.99 | 475.56±72.31 | 492.13±90.30 | 457.85±100.14 |
|  | RD | 422.67±90.42 | 444.08±73.89 | 489.30±89.51 | 452.05±120.71 | 437.49±81.95 |
|  | RI | 463.73±140.40 | 424.93±123.05 | 401.04±125.55 | 431.14±120.35 | 452.23±127.93 |
| Rotation duration  (ms) | TD | 342.92±71.00 | 363.11±91.37 | 369.30±95.16 | 371.21±88.73 | 380.41±76.32 |
|  | TI | 335.94±50.41 | 344.49±45.44 | 323.49±45.36 | 326.09±39.13 | 338.90±69.74 |
|  | RD | 374.63±67.32 | 433.04±77.36 | 425.32±68.76 | 372.81±88.44 | 351.18±56.02 |
|  | RI | 363.05±76.99 | 331.27±72.44 | 341.07±84.72 | 366.48±88.47 | 362.99±75.51 |
