# Supplementary material for: Different But Complementary Motor Functions Reveal an Asymmetric Recalibration of Upper Limb Bimanual Coordination
Source: eNeuro. 2026 Jan 2;13(1):ENEURO.0112-25.2025. doi: 10.1523/ENEURO.0112-25.2025 (PMC12794948; doi:10.1523/ENEURO.0112-25.2025)
Supplement: Figure 9-1 — Measurements of interlimb coordination at different experiment phases in Experiment 2. Summary of interlimb coordination measurements (max correlation and correlation lags) at different Experiment 2 phases (mean ± SEM) for the four participant groups. The results shown were not baseline subtracted. Download Figure 9-1, DOCX file. [file eneuro-13-ENEURO.0112-25.2025-s007.docx]

**Figure 9-1. Measurements of interlimb coordination at different experiment phases in Experiment 2.**

| **Coordination** | **Group** | **Experiment Phase (Mean ± SEM)** | | | | |
| --- | --- | --- | --- | --- | --- | --- |
|  |  | Baseline | Early perturb | Late perturb | Early decay | Late decay |
| Max correlation | TD | 39.44±5.32 | 45.63±5.82 | 44.13±3.84 | 38.51±4.69 | 41.64±5.87 |
|  | TI | 36.94±3.54 | 31.57±3.25 | 33.64±3.22 | 37.35±2.95 | 37.25±3.53 |
|  | RD | 39.93±3.83 | 37.37±5.96 | 39.36±3.88 | 36.81±4.66 | 39.38±2.56 |
|  | RI | 36.70±7.20 | 37.73±5.88 | 37.16±4.44 | 35.89±7.24 | 38.39±5.67 |
| Correlation  Lags  (ms) | TD | -111.34±38.44 | -100.61±61.99 | -121.66±59.18 | -92.33±49.92 | -108.65±33.10 |
|  | TI | -113.84±45.75 | -98.02±29.34 | -81.94±29.11 | -82.01±32.19 | -102.43±40.72 |
|  | RD | -87.10±50.49 | -67.87±57.51 | -72.61±34.74 | -80.68±35.82 | -100.61±38.11 |
|  | RI | -79.15±83.50 | -57.81±94.18 | -56.53±95.86 | -33.01±108.09 | -49.75±84.85 |
